# Peer victimization and adjustment: Moderation by perceptions of same-race and other-race classmates’ behavior

**DOI:** 10.1017/S0954579426101679

**Published:** 2026-07-17

**Authors:** Wendy Troop-Gordon, Julia R. Gordon, Karl Christensen

**Affiliations:** Human Development and Family Science, Auburn University, USA

**Keywords:** aggression, internalizing problems, peer victimization, same-race peers, social misfit hypothesis

## Abstract

Drawing on the “social misfit” hypothesis, this study tested whether peer victimization is more predictive of maladjustment when children have many same-race classmates who they view as low in aggression. Also accounted for were children’s race, the racial composition of the classroom, and children’s perceptions of other-race peers. Data were collected in the fall, winter, and spring of a school year from 1,424 4^th^ and 5^th^ grade children (*M*_age_ = 10.06; *SD*_age_ = 0.67; Range_age_ = 8.43–13.03; 683 girls; 741 boys; 39.61% Black; 60.39% White) in the Southeastern United States. Measures included children’s ratings of the aggressiveness of their participating Black and White classmates; teacher-reports of depressive symptoms, anxiety, and social withdrawal; and peer-reports of peer victimization, aggression, and prosocial behavior. Consistent with the social misfit hypothesis, when children viewed same-race classmates as low in aggression, peer victimization was more strongly associated with internalizing problems in the fall for Black children (*R* ^2^: 0.05–0.16) and with heightened anxiety in the spring for White children with many same-race classmates (*R* ^2^ = 0.09). Although some protective effects of viewing other-race classmates as aggressive emerged, associations with heightened anxiety (*R* ^2^: 0.06–0.09) underscored the need to promote positive interracial relationships to optimize children’s mental health.

Drawing on theories of person-context fit (Magnusson & Stattin, 1998; Thomas & Chess, 1977), investigators have applied the “social misfit hypothesis” to examine whether the peer context shapes children’s interpretations of, and socioemotional responses to, peer victimization. Researchers posit that when peer victimization is experienced as an outlier from contextual norms (i.e., a misfit), the targeted individual engages in characterological self-blame, leading to heightened psychological distress (Graham, 2006). Much of the work has focused on either: (a) the extent to which peer victimization or aggression is normative for the peer group or (b) the ethnic/racial makeup of the children’s classroom or school. Consistent with the social misfit hypothesis, peer victimization is more strongly associated with self-blame and internalizing distress when peer victimization and aggression are non-normative for the peer group (e.g., Gini et al., 2020; Gu et al., 2024) or when the victim is in the ethnic/racial majority (e.g., Bellmore et al., 2004; Robinson et al., 2021). One could surmise then that peer victimization may have its most deleterious outcomes when children have a high percentage of same-race classmates who they view as generally low in aggression.

The current work tests this proposition with some notable deviations from previous studies. First, rather than relying on objective assessments of group norms, this research focuses on children’s perceptions of their peers’ aggressiveness. Conceptually, it is children’s phenomenological experience of the peer group that most proximally shapes the meaning they place on their victimization experiences. Thus, this methodological shift provides a closer approximation to the application of the social misfit hypothesis when applied to the psychological distress of peer victimization than studies that have relied on more objective measures of contextual norms. Second, theoretically, peer victimization-distress associations diminish when the context allows for external attributions. Drawing on this, we test not only whether perceptions of low levels of aggression among same-race peers amplify relations between peer victimization and adjustment problems, but also whether associations are weaker when children have many other-race peers who they view as aggressive, as this would facilitate attributing victimization to classmates’ outgroup biases, racial prejudices, and discrimination.

## Peer victimization and the misfit hypothesis

Experiences of peer taunts, exclusion, and aggression (i.e., peer victimization) lead to immediate distress (Morrow et al., 2019b; Troop-Gordon & Erath, 2022) and when experienced repeatedly can have long-term effects on psychological and physical well-being (McDougall & Vaillancourt, 2015). In accordance with person-context fit theories (e.g., Magnusson & Stattin, 1998; Thomas & Chess, 1977), intrapersonal risk factors for maladjustment (e.g., problematic behaviors, conflict with peers) have more deleterious consequences in those contexts in which the behaviors or traits deviate from group norms. Thus, being a social misfit (e.g., being aggressive in a peer group in which such behavior is uncommon) amplifies the likelihood of interpersonal rebuke (e.g., rejection, exclusion; Wright et al., 1986). Although the “social misfit hypothesis” has been primarily applied to understanding how behavioral profiles may differentially lead to social maladjustment, in their seminal work, Bellmore et al. (2004) applied it to understanding how experiences in the peer group, specifically victimization, may lead to greater psychological maladjustment when experienced in contexts in which such treatment is unexpected. They reasoned that characterological self-blame (i.e., ascribing victimization to deficiencies in one’s own relatively immutable traits; Janoff-Bulman, 1979), one mechanism through which peer victimization increases risk for psychopathology (Chen & Graham, 2012; Graham et al., 2006; although see Perren et al., 2013, for an exception), is more likely in those contexts in which experiencing peer aggression would least be expected (i.e., in which being peer-victimized is a misfit to the group context; see Garandeau & Salmivalli, 2019, for a discussion of these processes as studied within the literature on the healthy context paradox).

A peer group in which aggression and peer victimization are non-normative is one such context. Consistent with this premise, children have been found to ascribe greater characterological self-blame for peer victimization in schools and classrooms where peer victimization is relatively infrequent (Morrow et al., 2019a; Schacter & Juvonen, 2015). Associations are also stronger between peer victimization and negative self-evaluations (Gu et al., 2024; Xiong et al., 2023) and internalizing problems (Bellmore et al., 2004; Gini et al., 2020; Gu et al., 2024; Morrow et al., 2019b; Xiong et al., 2023; Yun & Juvonen, 2020) in peer groups with lower levels of peer victimization and aggression. Moreover, recent longitudinal research by Laninga-Wijnen et al. (2025) documented stronger prospective associations between peer victimization and psychological problems in classrooms with lower levels of peer victimization.

Characterological self-blame for peer victimization, and accompanying psychosocial problems, is also expected to be higher when the peer context consists of predominantly same-race peers (Bellmore et al., 2004; Graham, 2006). Categorization of individuals by race emerges early in child development (Pauker et al., 2016), and children’s understanding of race advances in middle and late childhood as they develop increasing appreciation for the cultural, historic, and societal underpinnings of racial identity (Quintana, 1998). Racial ingroup favoritism also emerges early in childhood (Hughes et al., 2023) and is evident during middle childhood. For example, children progressively have a greater proportion of same-race than other-race friendships during the elementary school years (Aboud et al., 2003; see also Killen et al., 2022). This increase in racial awareness, unfortunately, is accompanied by outgroup bias, including by the late elementary school years minority youth’s experiences of discrimination and exclusion, often by other-race peers (see Brown, 2008).

Thus, although research on ethnic–racial identity development and intergroup relationships often focuses on adolescence, race has a substantial impact on self-understanding and peer relationships starting in childhood. The Developmental Subjective Group Dynamics (DSGD) model (Abrams & Rutland, 2008) points to the middle childhood years as a critical period for cognitive development that facilitates ingroup biases and leads to disapproval of ingroup members who deviate from ingroup norms and approval of outgroup members who deviate from outgroup norms. This theoretical framework also highlights how, due to advances in perspective taking during middle childhood, children begin to anticipate that others hold similar biases when evaluating ingroup and outgroup members. These maturing cognitive capacities and increasingly nuanced evaluations of ingroup and outgroup members likely have implications for children’s understanding of their own standing within their ingroup, including attributing within-group rejection as a failing to adhere to valued group expectations. Consonant with the social misfit hypothesis, then, negative treatment in contexts primarily composed of ingroup members, such as peers of one’s own race, should lead to greater self-blame and distress. In contrast, rejection from peer groups consisting primarily of outgroup members, such as other-race peers, may be attributed to adhering to norms of one’s ingroup and biases held by outgroup members, including prejudices due to racial identity.

Thus, by late childhood, being in the racial majority should likely amplify self-blame for peer victimization (i.e., “If I am the target of peers’ aggression when most of my peers are the same race as me, it must be something about me.”) and limit children’s ability to attribute peers’ aggression to external factors, namely other’s racial prejudice. In contrast, having predominantly other-race classmates could facilitate attributing peers’ aggression to racial bias and discrimination. Tests of this application of the social misfit hypothesis was pioneered by Graham and her colleagues (Bellmore et al., 2004; Graham et al., 2009) in their studies of adolescent development in the western U.S. Their research showed that peer victimization is more strongly associated with depression and low self-worth when youth are in the racial majority within their classroom and that this effect is partly due to higher levels of characterological self-blame.

Subsequent support for the misfit hypothesis has been found among Latino high school boys in the United States (U.S., Robinson et al., 2021) and Aboriginal adolescents in Canada (Hoglund & Hosan, 2013). In the only study to test this hypothesis among elementary school children, using the same data as the current study, Troop-Gordon et al. (2021) found that peer victimization was more strongly associated with low expectations of positive treatment from peers at higher levels of same-race classmates. However, these effects did not emerge for loneliness or anxiety. Furthermore, evidence of the misfit hypothesis as a function of peer group racial composition was not found in a study of sixth grade students in the U. S. (Mehari & Farrell, 2013) and has not been consistently supported across racial groups in previous studies (Hoglund & Hosan, 2013; Troop-Gordon et al., 2021). Thus, research is needed to understand the circumstances in which the peer group racial composition moderates the effects of peer victimization on well-being.

Taken together, theory and literature suggest that peer victimization may be: (a) most detrimental to the victim when the peer group is composed of same-race youth who the victim does not view as generally aggressive, leading the child to attribute peers’ aggression to one’s failure to meet the standards and expectations of one’s ingroup (i.e., self-blame) and (b) least detrimental when the peer group is comprised of many other-race peers who the victim views as generally aggressive to classmates, allowing them to attribute the victimization to discrimination and racism rather than to the self. The current study expands the literature by testing these premises. Specifically, data were used to test the interaction between peer victimization, the percentage of same-race classmates, and perceptions of the aggressiveness of both same-race and other-race classmates. Such research is timely as schools are experiencing shifts toward greater racial diversity (e.g., Juang & Schachner, 2020) or, as been happening in the U.S., greater resegregation (U.S. Government Accountability Office, 2022).

## Potential racial differences in misfit effects

To date, the application of the social misfit hypothesis to peer victimization-adjustment linkages has been predicated on the assumption that the influence of ethnic/racial classroom composition is similar across racial groups. However, the meaning of race within a classroom cannot be isolated from the meaning of race within the broader societal context. Historical, institutional, and structural power differentials across racial groups are deeply entrenched. Within the U.S., White racial identity is imbued with power and privilege, and minority racial groups, such as Black Americans, experience continued social, financial, and educational barriers. In the southern U.S. (Barber, 2023; Bartz & Kritsonis, 2019; Kihlström & Kirby, 2021; O’Connell, 2025) and in rural areas (Carter & Carter, 2014), the legacy and continuation of anti-Black racism can be deeply felt, including in the rural southern U.S. communities studied here.

Consequently, ethnic and racial identity are integral to child and adolescent development (Rivas-Drake et al., 2014). However, the intersection of race and developmental processes is not ubiquitous across racial groups. The centrality of race to one’s identity differs for Black and White children and can depend on context. Race is often a central part of Black children’s identity (Brown et al., 2011; Rowley et al., 2008; Ruble et al., 2004) due, in part, to socialization processes. For example, Black parents are often explicit and intentional in their socialization of racial identity (Hughes et al., 2008; Priest et al., 2014), and these socialization practices frequently begin when their children are young (Williams & Banarjee, 2021; Williams et al., 2017). Peers also play a prominent socializing role (Perry et al., 2025). Opportunities to spend time with same-race peers can heighten the centrality of race to Black youth’s identity, including increased racial pride (Santos et al., 2017), and having many same-race classmates may protect against race-based discrimination (Low et al., 2024).

Thus, even in contexts with many same-race peers, race may be a salient dimension with which Black children identify ingroup and outgroup members, anticipating greater similarity with, and acceptance from, other Black children. In contexts with many same-race peers, if aggressiveness is seen as a normative, Black children may interpret being victimized as a relatively typical experience rather than engaging in characterological self-blame, weakening associations with maladjustment. However, and consistent with the “social misfit” hypothesis, when Black children are in classrooms with many same-race peers who they view as low in aggression, peer victimization may likely be seen as non-normative and a reflection of lacking valued ingroup traits (Abrams & Rutland, 2008), leading to greater psychosocial maladjustment.

As race becomes more salient in heterogenous groups (Ruble et al., 2004), race likely remains, or becomes, even more salient for Black children when they have few same-race children. In such contexts, if other-race children are viewed as aggressive, Black children may readily attribute peer victimization to racial bias and prejudice. However, there is the possibility that the perceived aggressiveness of other-race classmates would have little effect on how peer victimization is interpreted by Black children when in classrooms with many other-race peers. By late childhood, Black children are often socialized to be alert for racial prejudice and, to a lesser degree, to mistrust those of other races (Hughes & Johnson, 2001; Hughes et al., 2023). The consequence may be that, even when other-race classmates are viewed as generally low in aggression, Black children may attribute peer victimization to racial prejudice. If this is the case, being in a classroom with a high percentage of same-race classmates may buffer associations between peer victimization and maladjustment for Black children regardless of how much they view other-race classmates as generally aggressive.

In contrast to Black children, race is typically not highly central to White children’s sense of self (Rogers & Meltzoff, 2017; Turner & Brown, 2007). This is due to both exposure to color-blind socialization practices that minimize race as a meaningful social category with which to understand human experiences (Abaied & Perry, 2021; Nieri et al., 2024), as well as prevailing White normativity within much of American society (Moffitt & Rogers, 2022). Furthermore, experiences of racial prejudice often serve as a catalyst for racial identity exploration (Quintana, 2007); yet, White children experience lower levels of race-based peer victimization than other children (Angus Reid Institute, 2021; Green et al., 2024). Rather, for White children, racial identity often becomes prominent only in contexts with greater racial diversity or in which White youth are in the racial minority (Phinney, 1991; Umaña-Taylor & Shin, 2007; Yip, 2008). Consequently, how classroom racial composition and perceptions of same-race and other-race peers may have different consequences for White children than for Black children. In classrooms with a large percentage of same-race classmates, race may not be a highly salient dimension with which White children identify ingroups and outgroups. White children in such classrooms may anticipate low levels of aggression, amplifying associations between peer victimization and maladjustment, but whether they view specifically their same-race peers as aggressive may not further moderate these effects. In classrooms with fewer same-race peers, race may become more salient to White children, including the possibility of being the target of aggression due to their race. Particularly if other-race peers are seen as aggressive, White children may attribute peer victimization to racial prejudices, buffering associations between peer victimization and maladjustment.

## The current study

Given the importance of understanding the experiences of Black and White children within classrooms of varying racial composition, the current study advances research on the social misfit hypothesis by testing whether a combination of perceived aggression of same-race and other-race classmates and racial composition of the classroom moderates associations between peer victimization and adjustment. The overarching hypotheses tested was that associations between peer victimization and maladjustment would be stronger when children had a large percentage of same-race classmates they viewed as nonaggressive and weaker if they had a large percentage of other-race classmates they viewed as aggressive. Data from Black and White children were analyzed separately to allow for the possibility that, for Black children, having a large percentage of other-race classmates (i.e., low levels of same-race classmates) would lead to weaker associations between peer victimization and adjustment regardless of how generally aggressive Black children view their other-race classmates and for the possibility that, for White children, having a large percentage of same-race classmates would lead to stronger associations between peer victimization and adjustment regardless of how aggressive White children viewed their same-race classmates.

Further, whereas most of the research testing the misfit hypothesis has relied on cross-sectional data (for exceptions, see Katulis et al., 2024; Laninga-Wijnen et al., 2025; Troop-Gordon et al., 2021), this study utilized longitudinal data tracking children across three time points within the same school year. The availability of longitudinal data allowed us to identify moderated effects in the fall and test whether they persisted throughout the school year, and to identify moderated effects that emerged during the school year possibly due to children experiencing increasing distress. Drawing on the DSGD model, we focus on late childhood, as this is a time in which perceptions of racial ingroups and outgroups should be emerging as an important factor in the socioemotional correlates of peer victimization, particularly as children spend the majority of their day with the same group of same-race and/or other-race peers.

The misfit hypothesis also has been primarily studied in relation to internalizing problems although evidence indicates that these same associations may also apply to externalizing problems (Katulis et al., 2024; Zhao & Li, 2022). The current study, therefore, examined a wide range of outcomes including internalizing problems (i.e., depression, anxiety, social withdrawal), externalizing problems (i.e., aggression), and peer-oriented behaviors (i.e., prosocial behavior). Prosocial behavior is a rather novel, but arguably important, addition to this literature. Peer victimization forecasts lower levels of prosocial behavior (Rudolph et al., 2014), potentially due to characterological self-blame and a depleted sense of self-efficacy to positively engage with others. Decreases in prosocial behavior can have downstream effects on children’s well-being. For example, lower levels of prosocial behavior in childhood are predictive of later internalizing and externalizing problems, as well as other clinical diagnoses (Eisenberg et al., 2024; Flynn et al., 2015), and poor academic achievement (Cruz et al., 2025; Gerbino et al., 2017; Li et al., 2026). Thus, identifying the factors that amplify or buffer against maladjustment resulting from peer victimization should not only include immediate risk of psychopathology but also disengagement from behaviors that foster mental health.

## Methods

### Participants

Participants included 1,424 4^th^ and 5^th^ grade children (683 girls; 564 Black; *M*_*age*_ = 10.06, *SD*_*age*_ = 0.67) from 13 public schools in rural communities in the Southeastern U.S. Data were collected in three waves over the course of the school year (Wave 1 = fall; Wave 2 = winter; Wave 3 = spring). The schools included 91 classrooms, and all classroom teachers consented to participate. All children were invited to participate with 1,564 (76.10%) receiving parental consent and providing written assent. Data from participating children who were not Black or White were excluded from analyses; thus, only data from students who were Black or White (91.05% of the recruited sample) were included. Children’s gender and race were obtained from school records. Participants came from primarily low-income households, and the schools served primarily low-income communities. The percentage of children at each school receiving reduced or free lunches ranged from 53.10% to 93.6%, (*M* = 71.31%). The teachers were predominantly White (78.90%; Black = 17.80%; Latina/o = 3.30%).

The vast majority of students (91.06%) in each participating classroom identified as Black or White, and in over 30% of the classrooms, only Black and White children participated. Notably, the racial distribution varied markedly from class to class (range Black: 5.30%–95.00%; range White: 0%–92.90%). Consequently, children whose race was not Black or White were highly underrepresented in this population and in the recruited sample (*n* = 140, 8.95%), and in all classrooms, the majority of students were either Black or White. Therefore, perceptions of only Black and White classmates were included in analyses.

### Measures

Means and standard deviations, presented separately for Black and White participants, and independent samples *t*-tests examining differences by race are presented in Table S1 in the supplement. Bivariate correlations are presented separately by race in Tables S2–S10 in the supplement.

#### Perceptions of Black and White classmates’ aggression

Children rated all of their participating classmates on three items assessing physical (“hit or push other kids”), verbal (“call other kids bad names or say mean things to them”), and relational aggression (“tell other kids they can’t play with them”) on a four-point scale (1 = *never*; 2 = *once or twice*; 3 = *sometimes*; 4 = *a lot*). The average rating children gave to their Black and White classmates in the fall was computed separately for each item, and item scores were averaged to create composite scores assessing perceived aggressiveness of Black classmates and perceived aggressiveness of White classmates (*α*_BlackClassmates_ **=** 0.83; *α*_WhiteClassmates_ **=** 0.82).

#### Percentage same-race classmates (PSRC)

Each child received a computed score that represented the percentage of classmates of the same race as the child. The number of children of each race (Black and White) in the classroom was summed and divided by the number of children in the class minus one. Children were assigned a PSRC score for their race.

#### Fall peer victimization

Children rated all participating classmates on three items assessing peer victimization [“get hit or pushed”; “gets left out of things that kids are doing (kids don’t let him or her play with them)”; “called bad names or say other mean things to him or her”] on a four-point scale (1 = *never*; 2 = *once or twice*; 3 = *sometimes*; 4 = *a lot*). For each item, the average rating received was computed, and these items scores were averaged to create a composite peer victimization score (*α* = 0.81).

#### Depression, anxiety, and social withdrawal

For each participating student, teachers completed six items from the TRF Anxious/Depressed subscale (Achenbach, 1991) measuring symptoms of depression (e.g., “feels worthless or inferior,” “feels or complains that no one loves him or her”), eight items from TRF Anxious/Depressed subscale measuring symptoms of anxiety (e.g., student is “afraid of making mistakes”; “worries”; or “self-conscious or easily embarrassed”), and six items from the Asocial with Peers subscale from the Child Behavior Scale (CBS-AS; Ladd & Profilet, 1996) measuring social withdrawal (e.g., “student prefers to play alone”; “keeps peers at a distance”; or “withdraws from peer activities”). Ratings were made on a three-point scale (1 = *not true as far as you know*; 2 = *somewhat or sometimes true*; 3 = *very true or often true*). Composite depression, anxiety, and social withdrawal scores were computed by averaging item scores. All measures had good internal reliability (αs_depression_ = 0.79, 0.82, and 0.81 and αs_anxiety_ = 0.86, 0.88, and 0.87, for fall, winter, and spring, respectively; αs_withdrawal_ = 0.92 at all waves).

#### Aggression

The ratings children received from all participating classmates on the three peer-rating items of aggression were averaged. Item scores were averaged to create a composite aggression score (*α* = 0.94, 0.94. and 0.93 for fall, winter, and spring, respectively).

#### Prosocial behavior

Children rated their participating classmates as to how often each “shares and acts nice” on a four-point scale (1 = *never*; 2 = *once or twice*; 3 = *sometimes*; 4 = *a lot*). This item is consistent with peer-report items of prosocial behavior used in the literature (e.g., Cillessen et al., 2005; Visconti & Troop-Gordon, 2010). Ratings received from participating classmates were averaged to create a composite prosocial behavior score.

### Procedures

Data were collected during two consecutive school years, in two separate cohorts. The first cohort consisted of five schools that participated during the 2017–2018 school year. The second cohort consisted of eight schools that participated during the 2018–2019 school year. Schools included fourth and fifth grade classes, except for one school that did not include fifth grade classes. All schools participated in a theory-based anti-bullying program with the focus of increasing defending behaviors when witnessing bullying. Prior to consent or data collection, schools were randomly assigned to either the intervention or control condition (54 intervention classrooms, 37 control classrooms; 91 classrooms total).

Data were collected at three separate waves during the school year (fall, winter, spring), with the intervention activity occurring after the fall winter data collection and before the winter data collection. Questionnaires were classroom-administered by trained undergraduate and graduate research assistants. One administrator read each question aloud, and one or more assistants helped the students as needed. The questionnaires took approximately 50–55 minutes to complete. The classes participated in one of two intervention activities. The first was based on Deviance Regulation Theory (DRT; Blanton & Burkley, 2008) and focused on increasing positive perceptions of defenders. The second focused on increasing empathy for victims. Both interventions were 45-minutes and included classroom discussion and a poster project. Inclusion of intervention condition did not change the pattern of results, and, therefore, was not included in the final analyses. This study was approved by the Institutional Review Board at Auburn University, Protocol #17-092 MR 1703, Project Title, “Using Deviance Regulation to Combat Bullying.”

### Missing data

Missing data ranged from 0.3% to 13.9% across all study variables. Comparisons of children with and without missing data revealed that Black children (38.7%) had more missing data than White children (19.70%), *χ* ^2^(1) = 62.14, *p* < .001. In addition, children with missing data perceived Black classmates as more aggressive than those without missing data and were reported to be more peer-victimized and aggressive, as well as less prosocial (Cohen’s *d* ranged from 0.20–0.38; see Table S11 in the supplement). Although children with missing data differed from those without missing data on a subset of study variables, these effects were small-to-moderate in magnitude.

### Analytic plan

Following an examination of the means, race and gender differences, bivariate correlations, and missing data, unconditional linear latent growth curves were estimated for each indicator of psychosocial adjustment using Mplus (Muthén & Muthén, 1998–2018) to assess mean change and the variance in the latent intercepts and slopes.

Conditional latent growth curves were then estimated separately for each indicator of psychosocial adjustment. As the goal of this work was to understand how peer victimization is associated with developmental processes for Black and White children, not to test whether processes were stronger for one group than the other, analyses were conducted separately by race. Each model included peer victimization, perceptions of Black classmates’ aggression, perceptions of White classmates’ aggression, and PSRC as predictors, as well as all two-way and three-way interactions between peer victimization, perceptions of Black classmates’ aggression, and PSRC and all two-way and three-way interactions between peer victimization, perceptions of White classmates’ aggression, and PSRC. Based on modification indices, also included in each model was the covariance between peer victimization × perceived aggression of White classmates × PSRC interaction and the peer victimization × perceived aggression of White classmates interaction and the covariance between perceived aggresion of White classmates and the perceived aggression of White classmates × PSRC interaction. The cluster option was used in all analyses to account for nesting within classroom. Independent samples *t*-tests examining gender differences in the study variables (see Table S12 in the supplement) revealed small to moderate differences (Cohen’s *d* ranged from 0.18–0.50), and the pattern of findings for the main analyses did not differ when gender was included as a covariate. Therefore, for parsimony, findings are presented without gender in the models.

Significant interactions were decomposed by calculating simple slopes at low (−1 *SD*) and high (+1*SD*) levels of the moderators and plotting trajectories at −1/+1 *SD* of peer victimization. We then recoded the latent slope from 0, 1, 2 to −2, −1, 0 to assess effects in the spring. We note effects that were marginally significant for descriptive purposes as weak effects may help explain why associations become statistically significant, or nonsignificant, in the spring. All models were estimated using full-information maximum likelihood (Enders & Bandalos, 2001), allowing for inclusion of data from all participants in model estimation. Model fit was assessed using the comparative fit index (CFI), the root mean square error of approximation (RMSEA), and the standardized root mean square residual (SRMR). Acceptable fit was indicated by CFI values ≥ 0.90, and RMSEA and SRMR values ≤ 0.08 (Hoe, 2008; Hu & Bentler, 1999).

## Results

### Unconditional models

The results of unconditional LGCMs estimated separately for Black and White children are presented in Table S13 and S14 in the supplement. On average, Black children evidenced a significant decrease in social withdrawal over the school year, and Black and White children evidenced increases in aggression. White children on average decreased in prosocial behavior. Across all analyses, there was significant variance in the latent intercept (all *ps* < .001) and in the latent slope (all *ps* ≤ .01) except for the slopes of depression and social withdrawal. For consistency, latent growth curve analyses were employed in all subsequent analyses.

### Prediction of socioemotional adjustment: Black children

The results of the LGCMs predicting Black children’s socioemotional adjustment are presented in Table 1. For ease of presentation, we summarize and present plotted trajectories only if they relate to moderation of the effects of peer victimization on the intercept or slope. Decomposition of interactions between PSRC and children’s perceptions of their same-race or other-race classmates can be found in the supplement. Confidence intervals for all parameters can be found in Tables S15–S24 in the supplement.

**Table 1.** Parameter estimates for latent growth curve models – Black children

Predictor	Dependent variable				
	Depression	Anxiety	Withdrawal	Aggression	Prosocial
Prediction of the intercept					
Peer victimization	0.23***	0.06	0.07	1.15***	−0.79***
PAG of Black classmates	−0.02	−0.01	−0.03	0.00	0.02
PAG of White classmates	0.01	−0.02	0.01	0.07^†^	−0.05
PSRC	−0.02	0.09	0.05	−0.12*	0.01
Peer victimization × PAG of Black classmates	−0.15*	−0.28**	−0.18*	−0.12	−0.03
Peer victimization × PAG of White classmates	0.12	0.31*	0.10	0.06	−0.02
Peer victimization × PSRC	−0.08	−0.19	−0.07	−0.48**	0.56*
PAG of Black classmates × PSRC	0.07	−0.04	−0.01	−0.09	0.30
PAG of White classmates × PSRC	−0.10	−0.01	−0.02	0.01	−0.08
PAG of Black classmates × Peer victimization × PSRC	0.26	−0.22	0.05	−0.55^†^	−0.24
PAG of White classmates × Peer victimization × PSRC	0.06	0.48	0.37	0.32	0.14
* R* ^2^	0.16	0.06	0.05	0.70	0.48
Prediction of the slope					
Peer victimization	−0.03^†^	0.01	0.01	−0.09**	−0.05
PAG of Black classmates	0.01	0.01	0.01	0.02	−0.05^†^
PAG of White classmates	0.01	0.02	0.02	−0.02	0.04
PSRC	−0.04	−0.10	−0.06	−0.03	0.15
Peer victimization × PAG of Black classmates	0.04	0.07*	0.08*	−0.03	0.09
Peer victimization × PAG of White classmates	−0.02	−0.08*	−0.05	0.02	0.02
Peer victimization × PSRC	−0.07	−0.05	0.02	−0.06	0.08
PAG of Black classmates × PSRC	−0.01	0.06	−0.05	0.00	−0.23*
PAG of White classmates × PSRC	0.01	−.07	0.06	0.07	0.11
PAG of Black classmates × Peer victimization × PSRC	0.00	0.05	0.15	0.08	0.10
PAG of White classmates × Peer victimization × PSRC	−0.07	0.03	−0.29	0.06	−0.31
*R* ^2^	0.12	0.08	0.29	0.07	0.11

*Note.* PAG = perceived aggression. PSRC = percentage same-race classmates.

^†^ *p* < .10. *p < .05. **p < .01. ***p < .001.

#### Depression

**Figure 1.**
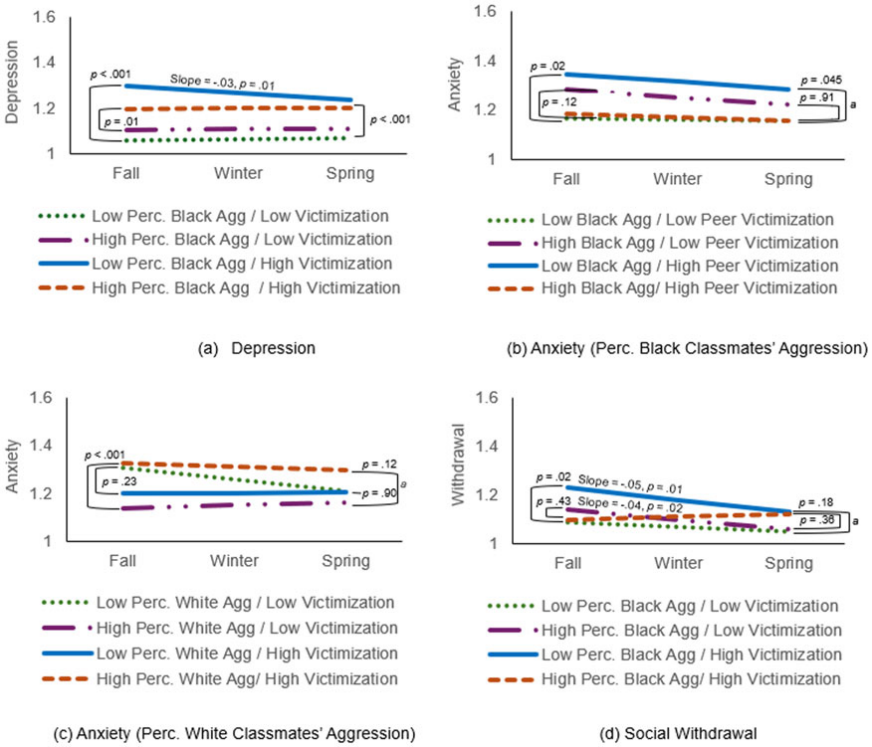
Estimated trajectories of depression, anxiety, and social withdrawal as a function of moderated peer victimization effects – Black children. *Note.* Perc. = perceived. Agg = aggression. ^a^ In the spring, the interaction was not significant, and the main effect of peer victimization was not significant. *P*-values represent the simple slopes of peer victimization at high and low levels of perceived classmates’ aggression; these are presented for descriptive purposes.

There was a significant negative peer victimization × perceived aggression of Black classmates interaction on the intercept. Peer victimization was more strongly associated with the intercept when Black children perceived their Black classmates as low in aggression (0.33, *p* < .001) than when they perceived their Black classmates as high in aggression (0.13, *p* = .01). Estimated trajectories are presented in Figure 1a. In the fall, the highest levels of depression were found when Black children experienced high levels of peer victimization and viewed their Black classmates as low in aggression. Although there were no significant predictors of the latent slope, the cumulation of predictive effects resulted in an estimated significant decrease in depression at high levels of peer victimization and low levels of perceived aggression among Black peers. By the spring, the interaction was no longer significant (−0.08, *p* = .22).

#### Anxiety

There was a significant negative peer victimization × perceived aggression of Black classmates interaction on the intercept and a positive peer victimization × perceived aggression of Black classmates interaction on the slope. Peer victimization was positively associated with the intercept when Black children viewed their Black peers as low in aggression (0.25, *p* = .02) and was not associated with the intercept when they perceived their Black classmates as high in aggression (−0.14, *p* = .12). Peer victimization was not associated with the slope when Black children viewed their Black classmates as low in aggression (−0.04, *p* = .35) and was marginally positively associated with the slope when they viewed their Black peers as high in aggression (0.06, *p* = .08). Estimated trajectories are presented in Figure 1b. In the fall, the highest levels of anxiety were found when Black children experienced high levels of peer victimization and viewed their Black classmates as low in aggression. Due to subtle differences in the slopes, by the spring, the interaction was no longer significant (−.014, *p* = .12).

There was a significant positive peer victimization × perceived aggression of White classmates interaction on the intercept and a marginally significant (*p* = .05) negative peer victimization × perceived aggression of White classmates interaction on the slope. Peer victimization was positively associated with the intercept when Black children perceived their White classmates as high in aggression (0.26, *p* = .01) and was not associated with the intercept when they perceived their White classmates as low in aggression (−0.15, *p* = .23). Peer victimization was not associated with the slope when Black children perceived their White classmates as high in aggression (−0.04, *p* = .35) and was marginally positively associated with slope when they viewed their White classmates as low in aggression (0.07, *p* = .08). Estimated trajectories are presented in Figure 1c. In the fall, the highest levels of anxiety were found when Black children experienced high levels of peer victimization and viewed their White classmates as high in aggression. Due to subtle differences in the slopes, by the spring, the interaction was no longer significant (0.15, *p* = .31).

#### Withdrawal

There was a marginally significant (*p* = .05) negative peer victimization × perceived aggression of Black classmates interaction on the intercept and a significant positive peer victimization × perceived aggression of Black classmates interaction on the slope. Peer victimization was positively associated with the intercept when Black children viewed their Black classmates as low in aggression (0.19, *p* = .02) but was not associated with the intercept when Black children viewed their Black classmates as low in aggression (−0.06, *p* = .43). Peer victimization was not associated with the slope when Black children viewed their Black classmates as high in aggression (−0.05, *p* = .15) and was marginally positively associated with the slope when they viewed their Black classmates as high in aggression (0.07, *p* = .06). Estimated trajectories are presented in Figure 1d. In the fall, the highest levels of withdrawal were found when Black children experienced high levels of peer victimization and perceived their Black classmates as low in aggression. Due to a significant decrease in withdrawal at high levels of peer victimization and low levels of perceived aggression among Black classmates, by the spring, the interaction was no longer significant (−0.02, *p* = .85).

#### Aggressive behavior

**Figure 2.**
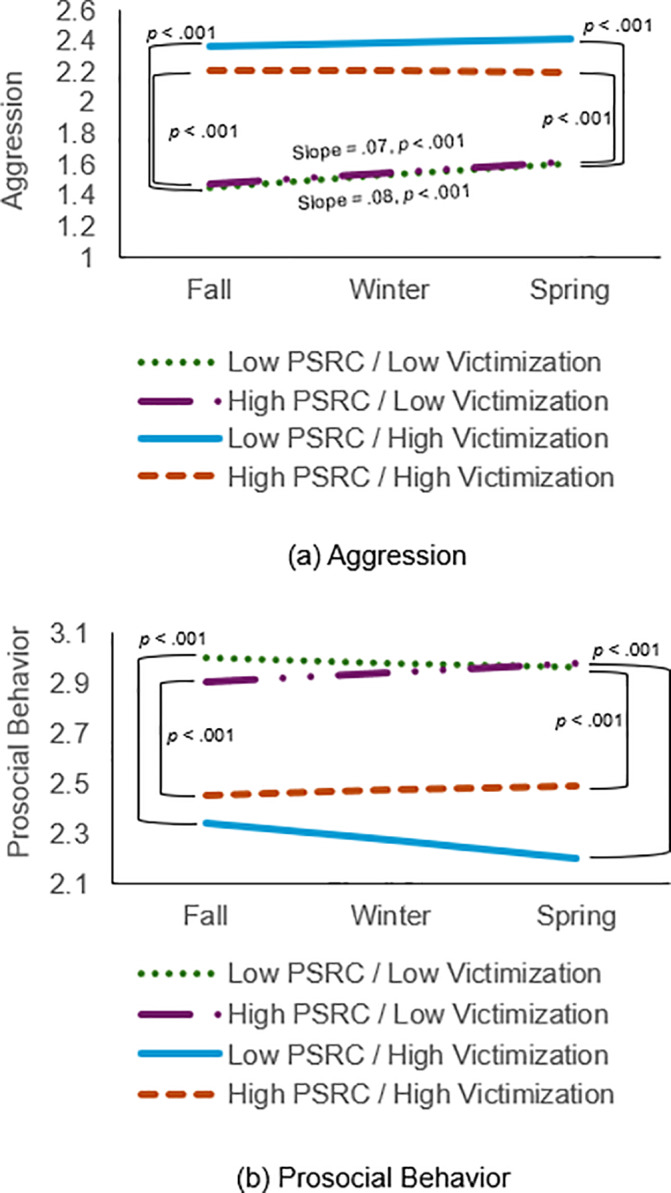
Estimated trajectories of aggression and prosocial behavior as a function of peer victimization moderated by percent same-race classmates – Black children. *Note.* PSRC = percentage same-race classmates.

There was a significant negative peer victimization × PSRC interaction on the intercept. Peer victimization was more strongly associated with the intercept when Black children had a low PSRC (1.28, *p* < .001) than when they had a high PSRC (1.02, *p* < .001). Estimated trajectories are presented in Figure 2a. In the fall, the highest levels of aggression were found when Black children experienced high levels of peer victimization and had a low PSRC. The interaction remained significant in the spring (−0.61, *p* = .045).

#### Prosocial behavior

There was a significant positive peer victimization × PSRC interaction on the intercept. Peer victimization was more strongly associated with the intercept when Black children had a low PSRC (−0.93, *p* < .001) than when they had a high PSRC (−0.64, *p* < .001). Estimated trajectories are presented in Figure 2b. In the fall, the lowest levels of prosocial behavior were found when Black children experienced high levels of peer victimization and had a low PSRC. The interaction was marginally significant in the spring (0.72, *p* = .055).

There was also a significant negative perceived aggression of Black classmates × PSRC interaction on the slope. Perceived aggression of black peers was negatively associated with the slope when Black children had a high PSRC (−0.11, *p* = .02) but was not associated with the slope when they had a low PSRC (0.01, *p* = .83). Estimated trajectories are presented in Figure S1 in the supplement. A nonsignificant (*p* = .09) increase in prosocial behavior was found when Black children had many same-race classmates who they viewed as low in aggression, but this increase did not lead to meaningful effects in the spring beyond the main negative effect of victimization.

### Prediction of socioemotional adjustment: White children

The results of the LGCMs predicting White children’s socioemotional adjustment are presented in Table 2. Confidence intervals for all parameters can be found in Table S16 in the supplement.

**Table 2.** Parameter estimates for latent growth curve models – White children

Predictor	Dependent variable				
	Depression	Anxiety	Withdrawal	Aggression	Prosocial
Prediction of the intercept					
Peer victimization	0.28***	0.06	0.18***	1.01***	−0.88***
PAG of White classmates	−0.01	−0.08	0.01	0.11***	0.01
PAG of Black classmates	−0.02	0.06	−0.01	−0.06**	0.03
PSRC	0.03	0.11	−0.09	0.10	0.14
Peer victimization × PAG of White classmates	0.15	0.05	−0.05	0.07	−0.16
Peer victimization × PAG of Black classmates	0.04	0.07	0.17	−0.22**	0.22*
Peer victimization × PSRC	−0.24	−0.37	−0.22	0.30	−0.94*
PAG of White classmates × PSRC	−0.48^†^	−0.26	0.21	−0.16	−0.37
PAG of Black classmates × PSRC	0.20	0.22	−0.17	0.04	−0.01
PAG of White classmates × Peer victimization × PSRC	0.69	0.01	0.31	0.75**	0.01
PAG of Black classmates × Peer victimization × PSRC	−0.09	0.27	−0.31	−0.93***	0.67*
*R* ^2^	0.20	0.03	0.10	0.69	0.49
Prediction of the slope					
Peer victimization	0.00	0.02	−0.01	−0.06*	−0.04
PAG of White classmates	0.00	0.03	0.00	−0.02	−0.05
PAG of Black classmates	0.02^†^	0.00	0.00	.03*	−0.03
PSRC	0.06	−0.01	−0.01	−0.02	−0.09
Peer victimization × PAG of White classmates	−0.04	−0.14**	0.01	−0.05	0.13
Peer victimization × PAG of Black classmates	0.03	0.13***	−0.03	0.02	−0.04
Peer victimization × PSRC	−0.05	0.11	0.01	0.11	0.33
PAG of White classmates × PSRC	0.20*	−0.10	−0.26^†^	−0.15	0.08
PAG of Black classmates × PSRC	−0.07	0.07	0.25**	0.03	0.20
PAG of White classmates × Peer victimization × PSRC	−0.42***	−0.51**	0.01	−0.14	−0.28
PAG of Black classmates × Peer victimization × PSRC	−0.03	0.08	0.19	0.25^†^	−0.54**
*R* ^2^	0.12	0.07	0.16	0.06	0.11

*Note.* PAG = perceived aggression. PSRC = percentage same-race classmates.

^†^ *p* < .10. *p < .05. **p < .01. ***p < .001.

#### Depression

There was a significant positive peer victimization × perceived aggression of White classmates × PSRC interaction on the slope. Peer victimization was marginally negatively associated with the slope when White children had a high PSRC who they viewed as high in aggression (−0.06, *p* = .07), but was not associated with the slope when they had a low PSRC who they viewed as low (−0.01, *p* = .87) or high (0.01, *p* = .57) in aggression or when they had a high PSRC who they viewed as low in aggression (0.04, *p* = .34). Estimated trajectories are presented in Figure S2 in the supplement. A significant increase in depression (0.04, *p* = .007) was found when White children experienced low levels of peer victimization and had a high PSRC who they viewed as high in aggression. This increase did not lead to meaningful effects in the spring beyond the positive main effect of peer victimization.

#### Anxiety

**Figure 3.**
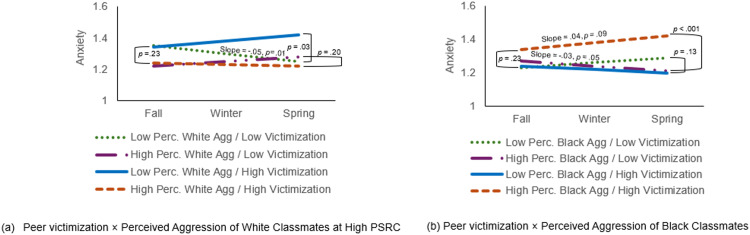
Estimated trajectories of anxiety as a function of moderated peer victimization effects – White children. *Note.* PSRC = percentage same-race classmates. Perc. = perceived. Agg = aggression.

There was a significant negative peer victimization × perceived aggression of White classmates × PSRC interaction on the slope. Peer victimization was positively associated with the slope when White children had a high PSRC who they viewed as low in aggression (0.14, *p* = .004), but was not associated with the slope when they had a high PSRC who they viewed as high in aggression (−0.07, *p* = .08) or a low PSRC who they viewed as low (0.03, *p* = .39) or high (−0.03, *p* = .42) in aggression. Estimated trajectories at a high PSRC are presented in Figure 3a (see Figure S3 for trajectories at a low PSRC). A significant decrease in anxiety was found when White children had low levels of peer victimization and viewed their White classmates as low in aggression. A nonsignificant increase in anxiety was found when White children had high levels of peer victimization and viewed their White classmates as low in aggression. By the spring, there was a significant peer victimization × perceived aggression of White classmates × PSRC interaction (−1.00, *p* = .02). Peer victimization was significantly associated with higher levels of anxiety when White children had a high PSRC who they viewed as low in aggression (0.25, *p* = .03) but was not associated with anxiety when they had a high PSRC who they viewed as high in aggression (−0.10, *p* = .20).

There was also a significant positive peer victimization × perceived aggression of Black classmates interaction on the slope. Peer victimization was positively associated with the slope when White children viewed their Black classmates as high in aggression (0.11, *p* = .001) but was negatively associated with the slope when they viewed their Black classmates as low in aggression (−0.07, *p* = .03). Estimated trajectories are presented in Figure 3b. Stable low levels of anxiety were found throughout the year with the exception that White children exhibited a marginal increase in anxiety when they reported high levels of peer victimization and viewed their Black classmates as high in aggression. By the spring, there was a significant peer victimization × perceived aggression of Black classmates interaction (0.33, *p* < .001). Peer victimization was significantly associated with higher levels of anxiety when White children viewed their Black classmates as high in aggression (0.31, *p* < .001) but not when they viewed them as low in aggression (−0.12, *p* = .13).

#### Withdrawal

There was a significant positive perceived aggression of Black classmates × PSRC interaction on the slope. Perceived aggression of Black classmates was positively associated with the slope when White children had a high PSRC (0.04, *p* = .005) but was negatively associated with the slope when White children had a low PSRC (−0.04, *p* = .02). Estimated trajectories are presented in Figure S4 in the supplement. Although there were differences in the estimated slopes, none of these slopes were significantly different from zero.

#### Aggressive behavior

**Figure 4.**
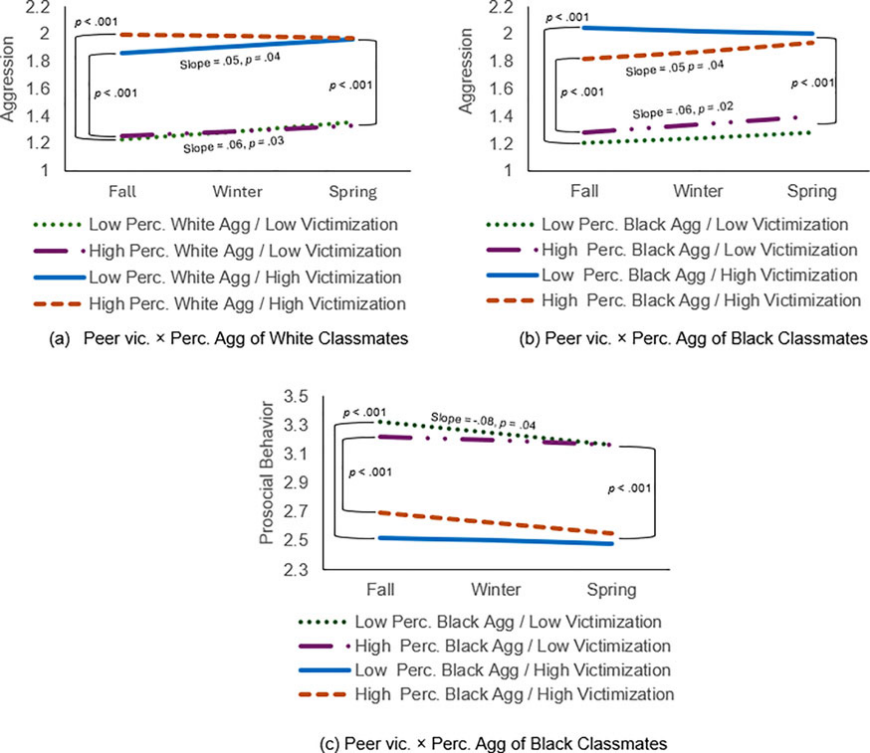
Estimated trajectories of aggression and prosocial behavior as a function of moderated peer victimization effects at a high PSRC – White children. *Note.* PSRC = percentage same-race classmates. Peer vic. = peer victimization. Perc. = perceived. Agg = aggression.

There was a positive peer victimization × perceived aggression of White classmates × PSRC interaction on the intercept. Although peer victimization was positively associated with aggressive behavior at all levels of the moderators, this effect was strongest when White children had a high PSRC who they view as high in aggression (high perceived White classmates’ aggressive behavior and low PSRC = 0.94, low perceived White classmates’ aggressive behavior and low PSRC = 0.98, high perceived White classmates’ aggressive behavior and high PSRC = 1.14, and low perceived White classmates’ aggressive behavior and high PSRC = 0.97, all *ps* < .001). Estimated trajectories at a high PSRC are presented in Figure 4a (see Figure S5 for trajectories at a low PSRC). At a high PSRC, in the fall, the highest levels of aggression were found when White children had high levels of peer victimization and viewed their White classmates as high in aggression. Due to subtle differences in the slopes, by the spring, the interaction was no longer significant (0.48, *p* = .18).

There was also a significant negative peer victimization × perceived aggression of Black classmates × PSRC interaction on the intercept and a marginally significant positive peer victimization × perceived aggression of Black classmates × PSRC interaction on the slope. Although peer victimization was positively associated with aggressive behavior in the fall at all levels of the moderators, this effect was strongest when White children viewed their Black classmates as low in aggression and had a high PSRC and lowest when White children viewed their Black classmates as high in aggression and had a high PSRC (low perceived Black classmates’ aggressive behavior and low PSRC = 1.02, high perceived Black classmates’ aggressive behavior and low PSRC = 0.91, low perceived Black classmates’ aggressive behavior and high PSRC = 1.28, and high perceived Black classmates’ aggressive behavior and high PSRC = 0.82, all *ps* < .001). Peer victimization was negatively, although weakly, associated with the slope at all levels of the moderators except when they viewed their Black classmates as high in aggression and had a high PSRC (low perceived Black classmates’ aggressive behavior and low PSRC = −0.07, *p* = .08; high perceived Black classmates’ aggressive behavior and low PSRC = −0.09, *p* = .02, low perceived Black classmates’ aggressive behavior and high PSRC = −0.09, *p* = .06, and high perceived Black classmate aggression and high PSRC = −0.01, *p* = .90). Estimated trajectories at a high PSRC are presented in Figure 4b (see Figure S6 in the supplement for trajectories at a low PSRC). At a high PSRC, the highest levels of peer-reported aggression were found when White children reported high levels of peer victimization and viewed their Black classmates as low in aggression. However, due to subtle differences in the slopes, the interaction was marginally significant in the spring (−0.44, *p* = .09).

#### Prosocial behavior

There was a significant positive peer victimization × perceived aggression of Black classmates × PSRC interaction on the intercept and a significant negative peer victimization × perceived aggression of Black classmates × PSRC interaction on the slope. Peer victimization was negatively associated with prosocial behavior in the fall at all levels of the moderator, but this association was strongest when White children had a high PSRC and viewed their Black classmates as low in aggression (low perceived Black classmates’ aggressive behavior and low PSRC = −0.81, high perceived Black classmates’ aggressive behavior and low PSRC = −0.66, low perceived Black classmates’ aggressive behavior and high PSRC = −1.22, and high perceived Black classmates’ aggressive behavior and high PSRC = −0.81, all *ps* < .001). Peer victimization was associated with a decline in prosocial behavior at all levels of the moderators except when White children had a high PSRC and viewed their Black classmates as low in aggression, although no slopes were significantly different from 0 (low perceived Black classmates’ aggressive behavior and low PSRC = −0.11, high perceived Black classmates’ aggressive behavior and low PSRC = −0.06, low perceived Black classmates’ aggressive behavior and high PSRC = 0.09, and high perceived Black classmates’ aggressive behavior and high PSRC = −0.07, all *ps* ≥ .21). Estimated trajectories at high PSRC are presented in Figure 4c (see Figure S7 in the supplement for trajectories at a low PSRC). At a high PSRC, the lowest levels of prosocial behavior were found when White children had high levels of peer victimization and viewed their Black classmates as low in aggression. Due to subtle differences in the slopes, the interaction was no longer significant in the spring (−0.42, *p* = .19).

## Discussion

As schools become increasingly diverse worldwide (Juang & Schachner, 2020), and yet increasingly racially segregated in some areas (U.S. Government Accountability Office, 2022), there is a need to understand how children’s social experiences unfold within the racial structure of their peer group. The current study utilized the social misfit hypothesis (Graham, 2006) to examine whether associations between peer victimization and adjustment are moderated by perceptions of same-race and other-race classmates’ aggressiveness. Several findings were consistent with the social misfit hypothesis, underscoring that peer victimization is more likely to predict internalizing problems when perceptions of the peer context do not readily allow for external attributions for the bullying. Perceptions of other-race peers also moderated associations, including results suggesting that viewing other-race peers as aggressive ampifies anxiety among children victimized by peers. Together, these results highlight the need to address children’s perceptions of their social environment, their racial identity, and the racial composition of the peer group when examining how peer victimization serves as a risk factor for psychopathology and poor social development.

### Moderation by perceptions of same-race peers

For Black children, perceptions of same-race peers moderated the effects of peer victimization for three out of the five outcome variables studied. Specifically, peer victimization was less strongly associated with depression, anxiety, and social withdrawal when Black children viewed their same-race classmates as high in aggression. By viewing their same-race classmates as aggressive, they may have been able to discount peer victimization as a normal part of peer interactions, at least among their Black classmates. In contrast, those viewing same-race classmates as low in aggression may have made characterological self-attributions for being bullied (Graham, 2006), leading to greater internalizing symptoms. These effects, however, were limited to the fall. It is possible that viewing same-race classmates as non-aggressive initially contributed to emotional distress, but over time, these positive perceptions promoted healthy peer interactions (Salmivalli et al., 2005) which, in turn, reduced peer victimization and internalizing problems. Alternatively, these peer-victimized children may have come to view their same-race peers as aggressive, reducing their self-blame and associated distress.

Inconsistent with our hypotheses, associations were, for the most part, not stronger for Black children when they had a larger number of same-race classmates. This suggests that, regardless of the racial composition of the classroom, for Black children, same-race classmates are the proximal reference group with which they form expectations for other’s behavior and interpret their social experiences. As racial identity tends to be highly salient for Black youth (Umaña-Taylor & Shin, 2007), it follows that their expectations for social experiences may be shaped foremost by the behaviors of their same-race peers. Furthermore, as peer relationships within elementary school classrooms tend to be somewhat racially segregated, interactions with Black classmates may constitute much of their social experiences even within classrooms with few same-race classmates (Wilson & Rodkin, 2011).

Perceptions of the aggression of same-race peers was a significant moderator in two analyses for White children. Peer victimization was associated with a trajectory of increasing anxiety over the school year when White children had many same-race classmates whom they viewed as low in aggression. It is difficult to discern why moderation was found for anxiety but not depression and social withdrawal. However, there is evidence that cognitive regulatory processes uniquely mediate associations between peer victimization and anxiety, but not depression (Adrian et al., 2019). It is possible then that when White children are in a classroom with same-race peers whom they view as low in aggression they increasingly make characterological self-attributions for their peer victimization, inhibiting the cognitive regulation necessary to lessen anxiety. Over time, these children may develop other internalizing problems such as depression and withdrawal.

Contrary to the social misfit hypothesis, peer victimization was associated with less aggression in the fall for White children when they had many same-race classmates who they viewed as infrequently aggressive. This protective effect was temporary, as aggression levels subsequently increased during the school year. We are only beginning to test the extent to which the misfit hypothesis extends to externalizing outcomes. There are reasons to believe that children who are peer-victimized may be at greater risk for aggression if they have many same-race classmates they view as aggressive. Viewing same-race classmates as aggressive, particularly when there are many of them, may contribute to holding broader schemas of the peer group as hostile, a known risk factor for externalizing problems (Ladd & Troop-Gordon, 2003; Salmivalli et al., 2005). Children who view aggression as normative among same-race classmates may also befriend peers whom they view as aggressive, exacerbating their own behavioral problems (Sijtsema et al., 2010). However, there are also reasons to expect that peer-victimized children would experience greater aggression when they view their same-race classmates as low in aggression. In healthy contexts, peer victimization may lead to feeling singled out, resulting in hostile attribution biases and reactive aggression (Liu et al., 2021). This explanation would account for the increase in aggressive behavior found among White children who had many same-race classmates they viewed as low in aggression. Understanding the specific cognitive mechanisms that account for aggression within specific classroom racial compositions, therefore, will be an important direction for future research.

### Moderation by perceptions of other-race peers

This study was novel in its examination of how perceptions of other-race classmates affect associations between peer victimization and adjustment. We posited that viewing other-race peers as aggressive facilitates making external attributions for peer victimization (Graham, 2006), weakening the effect of being bullied on well-being. Consistent with this proposition, for White children, peer victimization was less strongly associated with high levels of aggression and low levels of prosocial behavior when they viewed other-race peers as aggressive. However, this was true only in the fall and only when they had many White classmates. White children may be able to dismiss peer victimization if they can attribute it to the racial discrimination of a small number of classmates, reducing how much they act out against peers through aggression and low levels of prosocial behavior. However, if the victimization continues, particularly if they are in a class with primarily same-race classmates, White children’s ability to make external attributions for their victimization may diminish, leading them to adopt a more antisocial, less prosocial, orientation toward their peers.

Viewing other-race classmates as aggressive may also incur costs to well-being. In the current study, peer victimization was more strongly associated with anxiety for Black and White children when they viewed their other-race classmates as aggressive. Although such perceptions may facilitate making external attributions for peer victimization, they may not entirely serve as a protective factor. Racial discrimination in childhood is associated with greater anxiety and school avoidance (Anderson et al., 2020; Joo et al., 2023). Thus, when children view other-race classmates as aggressive, they may perceive the classroom context as less safe leading to heightened anxiety. This also may explain why peer victimization was more strongly associated with high levels of aggression and low levels of prosocial behavior for Black children when they had a high percentage of other-race classmates.

### Strengths, limitations, and future research directions

A substantial strength of the current research was the inclusion of longitudinal data from a large number of classrooms, allowing us to examine children’s perceptions of same-race and other-race classmates within classrooms that varied in their racial composition. While the social misfit hypothesis provided a framework for interpreting many of the findings, a limitation of this work is that the presumed mechanism, characterological self-blame, was not assessed. Plausible alternative explanations should be acknowledged and tested. As pointed out by Garandeau and Salmivalli (2019), in “healthy” contexts, children who are peer victimized may compare their peer experiences to classmates with more positive peer relationships, may have fewer opportunities to form friendships due to the absence of classmates who share their social difficulties, and may experience greater rejection due to being viewed by peers as responsible for their own bullying. Thus, characterological self-blame and these alternative explanations for social misfit effects need to be examined in future studies.

An additional strength of this research was the longitudinal design which allowed for determining which associations were transient (i.e., fall only), maintained (i.e., fall through spring), or emergent (i.e., spring-only). Few studies of the misfit hypothesis have examined prospective associations (see Gu et al., 2024; Katulis et al., 2024; Laninga-Wijnen et al., 2025), limiting our understanding of the temporal ordering of effects. For example, Garandeau and Salmivalli (2019) suggested that children who are peer-victimized in contexts that would presumably support healthy peer relationships may be those most at risk for maladjustment. Although this explanation could account for the concurrent associations found in this study, it does not explain some of the longitudinal findings, such as the increases in anxiety that were predicted for peer-victimized White children who viewed their same-race classmates as low in aggression.

Another strength of this study was the multi-informant design, incorporating self-report, peer-report, and teacher-report data. One limitation, however, was the unavailability of self-reports of depression and anxiety. Although well-validated measures were used, it is possible that findings would have been stronger if self-reported depression and anxiety had been assessed. Similarly, previous research has shown that self-reports of peer victimization are better predictors of internalizing problems than peer-reports (Graham & Juvonen, 1998). In addition, as is often the case, the data collected here did not allow for determining whether the classmates peer-victimized children rated as aggressive were classmates who bullied them. To more precisely investigate how racial dynamics impact peer victimization in the classroom and the effects of being bullied, research is needed in which bully-victim dyads are identified (see Rodkin et al., 2014, Veenstra et al., 2007, for examples). Furthermore, an analytic choice was made to examine data from Black and White children separately, potentially reducing power to identify main or interactive effects common to both groups. Collapsing data across racial groups would increase power and allow for testing novel research questions such as whether there are associations between the perceptions of children in the classroom’s racial majority and those in the racial minority. Thus, replication and extension of the current findings are needed using a variety of data sources, methodologies, and analytic strategies.

The findings are also specific to a particular geographical region, specific racial groups, and one developmental period. Race is culturally embedded (Seaton et al., 2006), and results drawn from Black and White children in the rural U.S. South may be different than those found in other countries or more urban communities. Furthermore, because the classrooms consisted of primarily Black and White children, we were unable to examine how children are affected by perceiving classmates of varying racial backgrounds as aggressive or nonaggressive. Examining how peer victimization is associated with adjustment for children in classrooms with varying racial compositions is an important direction for future research. In addition, adolescence is a critical period for racial identity development, and peer relationships become increasingly racially segregated as youth move into secondary schools (Kogachi & Graham, 2021). Thus, an important extension of the current study will be to examine how children’s perceptions of same-race and other-race peers moderate associations between peer victimization and adjustment in adolescence.

### Conclusions and clinical implications

Peer victimization is a highly stressful social experience that endangers children’s sense of self, trust in others, and mental health. Children’s efforts to make sense of mistreatment from peers are embedded within the racial composition of the peer group. However, as the current study demonstrates, it is not sufficient to solely account for the percentage of same-race classmates. Rather, children’s perceptions of their classmates play an equally significant role with peer victimization often being associated with more deleterious outcomes when peer-victimized children view their same-race classmates as low in aggression. At minimum, this research speaks to the need to identify children whose peer difficulties may go unnoticed due to being part of a racial majority, particularly if those children feel their bullying is an outlier among same-race peers. When working with peer-victimized youth, clinicians should address how children’s race and their perceptions of same-race and other-race peers inform their sense of self and understanding of their social experiences. Further, although viewing other-race classmates as aggressive may have some protective benefits for children who are peer victimized, the associations with heightened anxiety underscore that fostering positive interracial relationships has mental health, as well as societal, benefits.

## Supporting information

10.1017/S0954579426101679.sm001 Troop-Gordon et al. supplementary material Troop-Gordon et al. supplementary material

## Data Availability

Data are not available due to lack of permission from participating schools. Anonymized data are available from the corresponding author [WTG] upon reasonable request. All code can be found at https://osf.io/yrh9a/overview?view_only=ebedce65d81241e2b8aaf74cef06b382. All measures are available from the corresponding author [WTG] upon request.

## Supplementary material

The supplementary material for this article can be found at https://doi.org/10.1017/S0954579426101679.

## Pre-registration statement

This research and data analysis plan were not preregistered. The initial project was started in 2017, and the primary investigator was not provided training on registration at that time. These secondary analyses emerged as part of a student project to learn latent growth curve analysis. The decision to seek publication occurred after discovering that findings supported our hypotheses.
