# Supplementary material for: Peer victimization and adjustment: Moderation by perceptions of same-race and other-race classmates’ behavior
Source: Dev Psychopathol. 2026 Jul 17:1–16. Online ahead of print. doi: 10.1017/S0954579426101679 (PMC13423607; doi:10.1017/S0954579426101679)
Supplement: Troop-Gordon et al. supplementary material [file S0954579426101679sup001.docx]

Table S1

*Descriptive Statistics and Differences across Black and White Children*

|  | Black children | |  | White children | |  |  |  |
| --- | --- | --- | --- | --- | --- | --- | --- | --- |
| Variable | *M* | *SD* |  | *M* | *SD* |  | *t*-test | Cohen’s *d* |
| Perceived aggression – Black peers | 1.90 | .69 |  | 1.77 | .65 |  | 3.68^***^ | .21 |
| Perceived aggression – White peers | 1.64 | .66 |  | 1.57 | .48 |  | 2.19^*^ | .13 |
| Peer victimization | 1.65 | .36 |  | 1.52 | .33 |  | 6.87^***^ | .38 |
| PSRC | .46 | .26 |  | .62 | .15 |  | -14.55^***^ | -.79 |
| Fall depression | 1.16 | .28 |  | 1.17 | .29 |  | -.69 | -.04 |
| Winter depression | 1.17 | .30 |  | 1.17 | .31 |  | -.15 | -.01 |
| Spring depression | 1.15 | .27 |  | 1.17 | .30 |  | -1.44 | -.08 |
| Fall anxiety | 1.24 | .34 |  | 1.28 | .36 |  | -2.03 | -.11 |
| Winter anxiety | 1.26 | .36 |  | 1.28 | .39 |  | -.80 | -.05 |
| Spring anxiety | 1.21 | .30 |  | 1.28 | .38 |  | -3.55^***^ | -.20 |
| Fall social withdrawal | 1.13 | .34 |  | 1.16 | .35 |  | -1.23 | -.07 |
| Winter social withdrawal | 1.12 | .32 |  | 1.15 | .35 |  | -1.55 | -.09 |
| Spring social withdrawal | 1.09 | .27 |  | 1.15 | .35 |  | -3.34^***^ | -.19 |
| Fall aggression | 1.86 | .51 |  | 1.57 | .41 |  | 11.84^***^ | .65 |
| Winter aggression | 1.91 | .53 |  | 1.57 | .44 |  | 12.83^***^ | .71 |
| Spring aggression | 1.94 | .53 |  | 1.63 | .44 |  | 11.76^***^ | .66 |
| Fall prosocial behavior | 2.67 | .51 |  | 2.91 | .48 |  | -8.89^***^ | -.49 |
| Winter prosocial behavior | 2.69 | .49 |  | 2.91 | .50 |  | -7.89^***^ | -.44 |
| Spring prosocial behavior | 2.68 | .57 |  | 2.84 | .61 |  | -4.72^***^ | -.26 |

^*^ *p* < .05. ^**^ *p* < .01. ^***^ *p* < .001.

Table S2

*Bivariate Correlations between Perceived Aggression of Black and White Peers, Peer Victimization, PSRC, and Depression*

| Variable | 1 | 2 | 3 | 4 | 5 | 6 | 7 |
| --- | --- | --- | --- | --- | --- | --- | --- |
| 1. PA – Black peers | --- | .63^***^ | .17^***^ | -.08^*^ | .03 | .05 | .06 |
| 2. PA – White peers | .61^***^ | --- | .24^***^ | .00 | .07^*^ | .06 | .08^*^ |
| 3. Peer victimization | .15^***^ | .15^***^ | --- | -.08^*^ | .35^***^ | .28^***^ | .35^***^ |
| 4. PSRC | .12^**^ | .11^*^ | .23^***^ | --- | .00 | -.04 | .03 |
| 5. Fall depression | .02 | .07 | .34^***^ | .07 | --- | .61^***^ | .61^***^ |
| 6. Winter depression | .04 | .09 | .25^***^ | -.02 | .66^***^ | --- | .62^***^ |
| 7. Spring depression | .13^**^ | .18^**^ | .25^***^ | -.01 | .63^***^ | .63^***^ | --- |

*Note*. PA = perceived aggression. PSRC = percentage of same-race classmates. Values below the diagonal are for Black children, above the diagonal are for White children.

^*^ *p* < .05. ^**^ *p* < .01. ^***^ *p* < .001.

Table S3

*Bivariate Correlations between Perceived Aggression of Black and White Peers, Peer Victimization, PSRC, and Anxiety*

| Variable | 1 | 2 | 3 | 4 | 5 | 6 | 7 |
| --- | --- | --- | --- | --- | --- | --- | --- |
| 1. PA – Black peers | --- | .63^***^ | .17^***^ | -.08^*^ | .06 | .05 | .11^**^ |
| 2. PA – White peers | .61^***^ | --- | .24^***^ | .00 | -.01 | .00 | .07 |
| 3. Peer victimization | .15^***^ | .15^***^ | --- | -.08^*^ | .08^*^ | .03 | .14^***^ |
| 4. PSRC | .12^**^ | .11^*^ | .23^***^ | --- | .02 | .03 | -.01 |
| 5. Fall anxiety | -.01 | -.01 | .12^**^ | .09^*^ | --- | .60^***^ | .52^***^ |
| 6. Winter anxiety | .01 | .05 | .12^**^ | .06 | .63^***^ | --- | .58^***^ |
| 7. Spring anxiety | .04 | .08 | .11^*^ | -.05 | .52^***^ | .57^***^ | --- |

*Note*. PA = perceived aggression. PSRC = percentage of same-race classmates. Values below the diagonal are for Black children, above the diagonal are for White children.

^*^ *p* < .05. ^**^ *p* < .01. ^***^ *p* < .001.

Table S4

*Bivariate correlation between Perceived Aggression of Black and White Peers, Peer Victimization, PSRC, and Withdrawal*

| Variable | 1 | 2 | 3 | 4 | 5 | 6 | 7 |
| --- | --- | --- | --- | --- | --- | --- | --- |
| 1. PA – Black peers | --- | .63^***^ | .17^***^ | -.08^*^ | .04 | .01 | .04 |
| 2. PA – White peers | .61^***^ | --- | .24^***^ | .00 | .05 | .04 | .05 |
| 3. Peer victimization | .15^***^ | .15^***^ | --- | -.08^*^ | .21^***^ | .15^***^ | .19^***^ |
| 4. PSRC | .12^**^ | .11^*^ | .23^***^ | --- | -.06 | -.05 | -.05 |
| 5. Fall withdrawal | -.02 | .02 | .12^**^ | .06 | --- | .58^***^ | .57^***^ |
| 6. Winter withdrawal | .03 | .07 | .10^*^ | .02 | .48^***^ | --- | .59^***^ |
| 7. Spring withdrawal | .10^*^ | .17^***^ | .11^*^ | -.02 | .47^***^ | .51^***^ | --- |

*Note*. PA = perceived aggression. PSRC = percentage of same-race classmates. Values below the diagonal are for Black children, above the diagonal are for White children.

^*^ *p* < .05. ^**^ *p* < .01. ^***^ *p* < .001.

Table S5

*Bivariate Correlation between Perceived Aggression of Black and White Peers, Peer Victimization, PSRC, and Peer Reported Aggression*

| Variable | 1 | 2 | 3 | 4 | 5 | 6 | 7 |
| --- | --- | --- | --- | --- | --- | --- | --- |
| 1. PA – Black peers | --- | .63^***^ | .17^***^ | -.08^*^ | .10^**^ | .12^***^ | .13^***^ |
| 2. PA – White peers | .61^***^ | --- | .24^***^ | .00 | .23^***^ | .21^***^ | .19^***^ |
| 3. Peer victimization | .15^***^ | .15^***^ | --- | -.08^*^ | .79^***^ | .68^***^ | .63^***^ |
| 4. PSRC | .12^**^ | .11^*^ | .23^***^ | --- | -.01 | .00 | -.00 |
| 5. Fall aggression | .16^***^ | .19^***^ | .79^***^ | .13^**^ | --- | .82^***^ | .77^***^ |
| 6. Winter aggression | .18^***^ | .18^***^ | .71^***^ | .12^**^ | .85^***^ | --- | .85^***^ |
| 7. Spring aggression | .18^***^ | .12^*^ | .65^***^ | .07 | .80^***^ | .87^***^ | --- |

*Note*. PA = perceived aggression. PSRC = percentage of same-race classmates. Values below the diagonal are for Black children, above the diagonal are for White children.

^*^ *p* < .05. ^**^ *p* < .01. ^***^ *p* < .001.

Table S6

*Bivariate Correlation between Perceived Aggression of Black and White Peers, Peer Victimization, PSRC, and Peer Reported Prosocial Behavior*

| Variable | 1 | 2 | 3 | 4 | 5 | 6 | 7 |
| --- | --- | --- | --- | --- | --- | --- | --- |
| 1. PA – Black peers | --- | .63^***^ | .17^***^ | -.08^*^ | -.05 | -.14^***^ | -.13^**^ |
| 2. PA – White peers | .61^***^ | --- | .24^***^ | .00 | -.11^**^ | -.17^***^ | -.19^***^ |
| 3. Peer victimization | .15^***^ | .15^***^ | --- | -.08^*^ | -.56^***^ | -.58^***^ | -.49^***^ |
| 4. PSRC | .12^**^ | .11^*^ | .23^***^ | --- | .09^**^ | .01 | .02 |
| 5. Fall prosocial | -.08 | -.09 | -.54^***^ | -.15^***^ | --- | .64^***^ | .50^***^ |
| 6. Winter prosocial | -.15^***^ | -.08 | -.58^***^ | -.02 | .62^***^ | --- | .66^***^ |
| 7. Spring prosocial | -.12^**^ | -.03 | -.53^***^ | .02 | .49^***^ | .72^***^ | --- |

*Note*. PA = perceived aggression. PSRC = percentage of same-race classmates. Values below the diagonal are for Black children, above the diagonal are for White children.

^*^ *p* < .05. ^**^ *p* < .01. ^***^ *p* < .001.

Table S7

*Bivariate Correlations between Depression and all other Criterion Variables*

|  | Black children | | |  | White children | | |
| --- | --- | --- | --- | --- | --- | --- | --- |
| Variable | Fall Depression | Winter Depression | Spring Depression |  | Fall Depression | Winter Depression | Spring Depression |
| Fall anxiety | .58 | .43 | .39 |  | .54 | .35 | .32 |
| Winter anxiety | .47 | .62 | .43 |  | .35 | .55 | .38 |
| Spring anxiety | .41 | .42 | .64 |  | .40 | .38 | .58 |
| Fall withdrawal | .51 | .37 | .27 |  | .40 | .28 | .27 |
| Winter withdrawal | .38 | .54 | .36 |  | .24 | .44 | .30 |
| Spring withdrawal | .29 | .34 | .50 |  | .32 | .33 | .50 |
| Fall aggression | .25 | .18 | .17 |  | .30 | .23 | .30 |
| Winter aggression | .23 | .15 | .16 |  | .29 | .25 | .30 |
| Spring aggression | .21 | .13^**^ | .17 |  | .25 | .24 | .29 |
| Fall prosocial | -.17 | -.14^**^ | -.14^**^ |  | -.26 | -.20 | -.23 |
| Winter prosocial | -.23 | -.24 | -.21 |  | -.27 | -.23 | -.28 |
| Spring prosocial | -.14^**^ | -.10^*^ | -.06^NS^ |  | -.20 | -.18 | -.21 |

*Note.* All correlations are significant at *p* < .001 unless indicated otherwise. ^NS^ *p* = non-significant. ^*^ *p* < .05. ^**^ *p* < .01.

Table S8

*Bivariate Correlations between Anxiety and other Criterion Variables*

|  | Black children | | |  | White children | | |
| --- | --- | --- | --- | --- | --- | --- | --- |
| Variable | Fall Anxiety | Winter Anxiety | Spring Anxiety |  | Fall Anxiety | Winter Anxiety | Spring Anxiety |
| Fall withdrawal | .44 | .33 | .28 |  | .37 | .22 | .23 |
| Winter withdrawal | .39 | .53 | .36 |  | .21 | .30 | .18 |
| Spring withdrawal | .26 | .31 | .45 |  | .22 | .23 | .42 |
| Fall aggression | .02^NS^ | .05^NS^ | .05^NS^ |  | .00^NS^ | -.02^NS^ | .07^NS^ |
| Winter aggression | .02^NS^ | .02^NS^ | .01^NS^ |  | .01^NS^ | .03^NS^ | .08^*^ |
| Spring aggression | .04^NS^ | .05^NS^ | .06^NS^ |  | .03^NS^ | .03^NS^ | .09^*^ |
| Fall prosocial | -.02^NS^ | -.04^NS^ | -.08^NS^ |  | -.01^NS^ | .04^NS^ | -.04^NS^ |
| Winter prosocial | -.07^NS^ | -.09^NS^ | -.07^NS^ |  | -.01^NS^ | -.02^NS^ | -.10^**^ |
| Spring prosocial | -.05^NS^ | .01^NS^ | .01^NS^ |  | -.03^NS^ | .02^NS^ | -.08^*^ |

*Note.* All correlations are significant at *p* < .001 unless indicated otherwise.

^NS^ *p* = non-significant. ^*^ *p* < .05. ^**^ *p* < .01.

Table S9

*Bivariate Correlations between Withdrawal and All Other Criterion Variables*

|  | Black children | | |  | White children | | |
| --- | --- | --- | --- | --- | --- | --- | --- |
| Variable | Fall Withdrawal | Winter Withdrawal | Spring Withdrawal |  | Fall Withdrawal | Winter Withdrawal | Spring Withdrawal |
| Fall aggression | .01^NS^ | -.01^NS^ | -.01^NS^ |  | .08^*^ | .03^NS^ | .08^*^ |
| Winter aggression | .00^**^ | -.03^NS^ | -.02^NS^ |  | .07^*^ | .05^NS^ | .03^NS^ |
| Spring aggression | .01^**^ | .00^NS^ | .02^NS^ |  | .07^*^ | .07^*^ | .06^NS^ |
| Fall prosocial | .00^NS^ | -.01^NS^ | .03^NS^ |  | -.06^NS^ | -.06^NS^ | -.08^*^ |
| Winter prosocial | -.04^NS^ | -.03^NS^ | .03^NS^ |  | -.12 | -.10^**^ | -.10^**^ |
| Spring prosocial | .00^NS^ | .04^NS^ | .07^NS^ |  | -.12^**^ | -.08^*^ | -.11^**^ |

*Note.* All correlations are significant at *p* < .001 unless indicated otherwise. ^NS^ *p* = non-significant. ^*^ *p* < .05. ^**^ *p* < .01.

Table S10

*Bivariate Correlations between Peer Reported Aggression and all other Criterion Variables*

|  | Black children | | |  | White children | | |
| --- | --- | --- | --- | --- | --- | --- | --- |
| Variable | Fall Aggression | Winter Aggression | Spring Aggression |  | Fall Aggression | Winter Aggression | Spring Aggression |
| Fall prosocial | -.66 | -.64 | -.58 |  | -.66 | -.59 | -.57 |
| Winter prosocial | -.64 | -.74 | -.69 |  | -.64 | -.73 | -.66 |
| Spring prosocial | -.55 | -.63 | -.65 |  | -.52 | -.58 | -.64 |

*Note.* All correlations are significant at *p* < .001 unless indicated otherwise. ^NS^ *p* = non-significant. ^*^ *p* < .05. ^**^ *p* < .01.

Table S11

*Independent T-tests Comparing Children with and without Missing Data*

|  | Missing | |  | Not Missing | |  |  |  |
| --- | --- | --- | --- | --- | --- | --- | --- | --- |
| Variable | *M* | *SD* |  | *M* | *SD* |  | *t*-test | Cohen’s *d* |
| Perceived aggression – Black peers | 1.93 | .65 |  | 1.79 | .67 |  | -3.01^**^ | -.20 |
| Perceived aggression – White peers | 1.66 | .62 |  | 1.58 | .53 |  | -1.92 | -.15 |
| Peer victimization | 1.67 | .38 |  | 1.54 | .32 |  | -6.28^***^ | -.38 |
| PSRC | .56 | .23 |  | .55 | .21 |  | -.88 | -.05 |
| Fall depression | 1.19 | .30 |  | 1.16 | .28 |  | -1.53 | -.10 |
| Winter depression | 1.17 | .28 |  | 1.17 | .31 |  | .13 | .01 |
| Spring depression | 1.18 | .29 |  | 1.16 | .29 |  | -1.12 | -.08 |
| Fall anxiety | 1.28 | .37 |  | 1.25 | .35 |  | -1.02 | -.07 |
| Winter anxiety | 1.27 | .37 |  | 1.27 | .38 |  | .08 | .01 |
| Spring anxiety | 1.27 | .34 |  | 1.25 | .36 |  | -1.13 | -.08 |
| Fall social withdrawal | 1.17 | .35 |  | 1.14 | .35 |  | -1.07 | -.07 |
| Winter social withdrawal | 1.15 | .33 |  | 1.14 | .34 |  | -.41 | -.03 |
| Spring social withdrawal | 1.16 | .35 |  | 1.12 | .32 |  | -1.54 | -.11 |
| Fall aggression | 1.79 | .52 |  | 1.64 | .45 |  | -5.20^***^ | -.32 |
| Winter aggression | 1.82 | .55 |  | 1.67 | .48 |  | -4.88^***^ | -.31 |
| Spring aggression | 1.86 | .53 |  | 1.72 | .49 |  | -4.40^***^ | -.28 |
| Fall prosocial behavior | 2.70 | .50 |  | 2.87 | .50 |  | 5.85^***^ | .36 |
| Winter prosocial behavior | 2.68 | .53 |  | 2.87 | .49 |  | 6.00^***^ | .38 |
| Spring prosocial behavior | 2.65 | .59 |  | 2.82 | .60 |  | 4.45^***^ | .29 |

^*^ *p* < .05. ^**^ *p* < .01. ^***^ *p* < .001.

Table S12

*Descriptive Statistics and Differences by Gender*

|  | Boys | |  | Girls | |  |  |  |
| --- | --- | --- | --- | --- | --- | --- | --- | --- |
| Variable | *M* | *SD* |  | *M* | *SD* |  | *t*-test | Cohen’s *d* |
| Perceived aggression – Black peers | 1.90 | .69 |  | 1.77 | .65 |  | 3.68^***^ | .21 |
| Perceived aggression – White peers | 1.64 | .66 |  | 1.57 | .48 |  | 2.19^*^ | .13 |
| Peer victimization | 1.65 | .36 |  | 1.52 | .33 |  | 6.87^***^ | .38 |
| PSRC | .46 | .26 |  | .62 | .15 |  | -14.55^***^ | -.79 |
| Fall depression | 1.16 | .28 |  | 1.17 | .29 |  | -.69 | -.04 |
| Winter depression | 1.17 | .30 |  | 1.17 | .31 |  | -.15 | -.01 |
| Spring depression | 1.15 | .27 |  | 1.17 | .30 |  | -1.44 | -.08 |
| Fall anxiety | 1.24 | .34 |  | 1.28 | .36 |  | -2.03 | -.11 |
| Winter anxiety | 1.26 | .36 |  | 1.28 | .39 |  | -.80 | -.05 |
| Spring anxiety | 1.21 | .30 |  | 1.28 | .38 |  | -3.55^***^ | -.20 |
| Fall social withdrawal | 1.13 | .34 |  | 1.16 | .35 |  | -1.23 | -.07 |
| Winter social withdrawal | 1.12 | .32 |  | 1.15 | .35 |  | -1.55 | -.09 |
| Spring social withdrawal | 1.09 | .27 |  | 1.15 | .35 |  | -3.34^***^ | -.19 |
| Fall aggression | 1.86 | .51 |  | 1.57 | .41 |  | 11.84^***^ | .65 |
| Winter aggression | 1.91 | .53 |  | 1.57 | .44 |  | 12.83^***^ | .71 |
| Spring aggression | 1.94 | .53 |  | 1.63 | .44 |  | 11.76^***^ | .66 |
| Fall prosocial behavior | 2.67 | .51 |  | 2.91 | .48 |  | -8.89^***^ | -.49 |
| Winter prosocial behavior | 2.69 | .49 |  | 2.91 | .50 |  | -7.89^***^ | -.44 |
| Spring prosocial behavior | 2.68 | .57 |  | 2.84 | .61 |  | -4.72^***^ | -.26 |

^*^ *p* < .05. ^**^ *p* < .01. ^***^ *p* < .001.

Table S13

*Unconditional Latent Growth Curve Models*

Parameter Estimates:

|  | Black children | | | |  | White children | | | | | |
| --- | --- | --- | --- | --- | --- | --- | --- | --- | --- | --- | --- |
| Outcome | Mean Intercept | Mean Slope | Variance Intercept | Variance Slope | |  | Mean Intercept | Mean Slope | Variance Intercept | Variance Slope |  |
| Depression | 1.16 | -.006 | .060^***^ | .004 | |  | 1.17 | .002 | .057^***^ | .002 |  |
| Anxiety | 1.24 | -.014 | .097^***^ | .014^†^ | |  | 1.28 | .001 | .099^***^ | .014^*^ |  |
| Withdrawal | 1.14 | -.021^*^ | .061^***^ | .002 | |  | 1.16 | -.004 | .073^***^ | .002 |  |
| Aggression | 1.87 | .042^**^ | .244^***^ | .021^**^ | |  | 1.57 | .034^**^ | .160^***^ | .018^***^ |  |
| Prosocial Behavior | 2.68 | .000 | .170^***^ | .038^**^ | |  | 2.92 | -.033 | .158^***^ | .030^**^ |  |

Fit Statistics

|  | Black children | | | |  | White children | | | |
| --- | --- | --- | --- | --- | --- | --- | --- | --- | --- |
| Outcome | χ^2^(1) | CFI | RMSEA | SRMR |  | χ^2^(1) | CFI | RMSEA | SRMR |
| Depression | .36 | 1.00 | .000 | .007 |  | .06 | 1.00 | .000 | .002 |
| Anxiety | 7.62 | .982 | .109 | .034 |  | .04 | 1.00 | .000 | .003 |
| Withdrawal | .23 | 1.00 | .000 | .005 |  | .10 | 1.00 | .000 | .003 |
| Aggression | .05 | 1.00 | .000 | .002 |  | 4.07 | .994 | .060 | .017 |
| Prosocial Behavior | .50 | 1.00 | .000 | .012 |  | 1.45 | .982 | .023 | .019 |

^†^*p* < .10. ^*^*p* < .05. ^**^*p* < .01. ^***^*p* < .001.

Table S14

*Fit Statistics for Conditional Latent Growth Curve Models*

|  | Black children | | | |  | White children | | | |
| --- | --- | --- | --- | --- | --- | --- | --- | --- | --- |
| Outcome | χ^2^(*df =*12) | CFI | RMSEA | SRMR |  | χ^2^ (*df =*12) | CFI | RMSEA | SRMR |
| Depression | 17.632 | .989 | .029 | .012 |  | 18.686 | .990 | .025 | .009 |
| Anxiety | 15.425 | .990 | .023 | .009 |  | 22.791 | .980 | .032 | .010 |
| Withdrawal | 12.405 | .998 | .008 | .012 |  | 20.545 | .984 | .029 | .012 |
| Aggression | 6.193 | 1.00 | .000 | .005 |  | 16.160 | .998 | .020 | .007 |
| Prosocial Behavior | 10.513 | 1.00 | .000 | .010 |  | 14.852 | .991 | .017 | .008 |

Table S15

*Parameter Estimates and 95% Confidence Intervals for Depression Latent Growth Curve Model – Black Children*

|  | Depression intercept | |  | Depression slope | |
| --- | --- | --- | --- | --- | --- |
| Predictor | *b* | 95% CI |  | *b* | 95% CI |
| Peer victimization | .23^***^ | .13, .33 |  | -.03^†^ | -.05, .00 |
| PAG of Black classmates | -.02 | -.07, .07 |  | .01 | -.01, .03 |
| PAG of White classmates | .01 | -.05, .07 |  | .01 | -.01, .04 |
| PSRC | -.02 | -.26, .22 |  | -.04 | -.10, .03 |
| Peer victimization × PAG of Black classmates | -.15^*^ | -.27, -.03 |  | .04 | -.01, .08 |
| Peer victimization × PAG of White classmates | .12 | -.07, .31 |  | -.02 | -.07, .03 |
| Peer victimization × PSRC | -.08 | -.41, .25 |  | -.07 | -.18, .05 |
| PAG of Black classmates × PSRC | .07 | -.11, .26 |  | -.01 | -.11, .09 |
| PAG of White classmates × PSRC | -.10 | -.40, .19 |  | .01 | -.11, .14 |
| PAG of Black classmates × Peer victimization × PSRC | .26 | -.40, .93 |  | .00 | -.21, .22 |
| PAG of White classmates × Peer victimization × PSRC | .06 | -.67, .79 |  | -.07 | -.33, .19 |

*Note.* PAG = perceived aggression. PSRC = percentage same-race classmates.

^†^*p* < .10. ^*^ *p* < .05. ^**^ *p* < .01. ^***^ *p* < .001.

Table S16

*Parameter Estimates and 95% Confidence Intervals for Anxiety Latent Growth Curve Model – Black Children*

|  | Anxiety intercept | |  | Anxiety slope | |
| --- | --- | --- | --- | --- | --- |
| Predictor | *b* | 95% CI |  | *b* | 95% CI |
| Peer victimization | .06 | -.08, .19 |  | .01 | -.04, .07 |
| PAG of Black classmates | -.01 | -.08, .05 |  | .01 | -.02, .04 |
| PAG of White classmates | -.02 | -.09, .06 |  | .02 | -.02, .06 |
| PSRC | .09 | -.21, .40 |  | -.10 | -.20, .01 |
| Peer victimization × PAG of Black classmates | -.28^**^ | -.48, -.08 |  | .07^*^ | .01, .14 |
| Peer victimization × PAG of White classmates | .31^*^ | .05, .58 |  | -.08^*^ | -.16, .00 |
| Peer victimization × PSRC | -.19 | -.73, .36 |  | -.05 | -.28, .18 |
| PAG of Black classmates × PSRC | -.04 | -.31, .23 |  | .06 | -.06, .18 |
| PAG of White classmates × PSRC | -.01 | -.41, .39 |  | -.07 | -.24, .10 |
| PAG of Black classmates × Peer victimization × PSRC | -.22 | -1.13, .68 |  | .05 | -.25, .36 |
| PAG of White classmates × Peer victimization × PSRC | .48 | -.34, 1.30 |  | .03 | -.30, .37 |

*Note.* PAG = perceived aggression. PSRC = percentage same-race classmates.

^†^*p* < .10. ^*^ *p* < .05. ^**^ *p* < .01. ^***^ *p* < .001.

Table S17

*Parameter Estimates and 95% Confidence Intervals for Social Withdrawal Latent Growth Curve Model – Black Children*

|  | Social withdrawal intercept | |  | Social withdrawal slope | |
| --- | --- | --- | --- | --- | --- |
| Predictor | *b* | 95% CI |  | *b* | 95% CI |
| Peer victimization | .07 | -.03, .16 |  | .01 | -.02, .05 |
| PAG of Black classmates | -.03 | -.08, .02 |  | .01 | -.01, .03 |
| PAG of White classmates | .01 | -.07, .09 |  | .02 | -.01, .06 |
| PSRC | .05 | -.16, .26 |  | -.06 | -.15, .03 |
| Peer victimization × PAG of Black classmates | -.18^*^ | -.36, .00 |  | .08^*^ | .00, .16 |
| Peer victimization × PAG of White classmates | .10 | -.12, .32 |  | -.05 | -.13, .02 |
| Peer victimization × PSRC | -.07 | -.48, .33 |  | .02 | -.12, .16 |
| PAG of Black classmates × PSRC | -.01 | -.23, .22 |  | -.05 | -.14, .04 |
| PAG of White classmates × PSRC | -.02 | -.33, .30 |  | .06 | -.09, .21 |
| PAG of Black classmates × Peer victimization × PSRC | .05 | -.89, .99 |  | .15 | -.21, .51 |
| PAG of White classmates × Peer victimization × PSRC | .37 | -.59, 1.32 |  | -.29 | -.66, .07 |

*Note.* PAG = perceived aggression. PSRC = percentage same-race classmates.

^†^*p* < .10. ^*^ *p* < .05. ^**^ *p* < .01. ^***^ *p* < .001.

Table S18

*Parameter Estimates and 95% Confidence Intervals for Aggressive Behavior Latent Growth Curve Model – Black Children*

|  | Aggressive behavior intercept | |  | Aggressive behavior slope | |
| --- | --- | --- | --- | --- | --- |
| Predictor | *b* | 95% CI |  | *b* | 95% CI |
| Peer victimization | 1.15^***^ | 1.06, 1.24 |  | -.09^**^ | -.14, -.03 |
| PAG of Black classmates | .00 | -.08, .08 |  | .02 | -.01, .05 |
| PAG of White classmates | .07^†^ | -.01, .15 |  | -.02 | -.06, .01 |
| PSRC | -.12^*^ | -.23, -.003 |  | -.03 | -.14, .07 |
| Peer victimization × PAG of Black classmates | -.12 | -.27, .03 |  | -.03 | -.12, .06 |
| Peer victimization × PAG of White classmates | .06 | -.14, .25 |  | .02 | -.09, .13 |
| Peer victimization × PSRC | -.48^**^ | -.84, -.13 |  | -.06 | -.30, .17 |
| PAG of Black classmates × PSRC | -.09 | -.35, .17 |  | .00 | -.12, .12 |
| PAG of White classmates × PSRC | .01 | -.31, .32 |  | .07 | -.07, .22 |
| PAG of Black classmates × Peer victimization × PSRC | -.55^†^ | -1.19, .09 |  | .08 | -.36, .52 |
| PAG of White classmates × Peer victimization × PSRC | .32 | -.39, 1.03 |  | .06 | -.45, .56 |

*Note.* PAG = perceived aggression. PSRC = percentage same-race classmates.

^†^*p* < .10. ^*^ *p* < .05. ^**^ *p* < .01. ^***^ *p* < .001 .

Table S19

*Parameter Estimates and 95% Confidence Intervals for Prosocial Behavior Latent Growth Curve Model – Black Children*

|  | Prosocial behavior intercept | |  | Prosocial behavior slope | |
| --- | --- | --- | --- | --- | --- |
| Predictor | *b* | 95% CI |  | *b* | 95% CI |
| Peer victimization | -.79^***^ | -.91, -.66 |  | -.05 | -.16, .07 |
| PAG of Black classmates | .02 | -.07, .11 |  | -.05^†^ | -.11, .001 |
| PAG of White classmates | -.05 | -.14, .04 |  | .04 | -.02, .10 |
| PSRC | .01 | -.28, .30 |  | .15 | -.07, .38 |
| Peer victimization × PAG of Black classmates | -.03 | -.21, .15 |  | .09 | -.05, .21 |
| Peer victimization × PAG of White classmates | -.02 | -.22, .19 |  | .02 | -.16, .20 |
| Peer victimization × PSRC | .56^*^ | .02, 1.10 |  | .08 | -.35, .51 |
| PAG of Black classmates × PSRC | .30 | -.06, .66 |  | -.23^*^ | -.45, -.01 |
| PAG of White classmates × PSRC | -.08 | -.40, .25 |  | .11 | -.11, .33 |
| PAG of Black classmates × Peer victimization × PSRC | -.24 | -.98, .50 |  | .10 | -.45, .65 |
| PAG of White classmates × Peer victimization × PSRC | .14 | -.66, .94 |  | -.31 | -.87, .26 |

*Note.* PAG = perceived aggression. PSRC = percentage same-race classmates.

^†^*p* < .10. ^*^ *p* < .05. ^**^ *p* < .01. ^***^ *p* < .001

Table S20

*Parameter Estimates and 95% Confidence Intervals for Depression Latent Growth Curve Model – White Children*

|  | Depression intercept | |  | Depression slope | |
| --- | --- | --- | --- | --- | --- |
| Predictor | *b* | 95% CI |  | *b* | 95% CI |
| Peer victimization | .28^***^ | .19, .37 |  | .00 | -.05, .04 |
| PAG of White classmates | -.01 | -.07, .05 |  | .00 | -.03, .02 |
| PAG of Black classmates | -.02 | -.06, .03 |  | .02^†^ | -.002, .03 |
| PSRC | .03 | -.20, .26 |  | .06 | -.01, .13 |
| Peer victimization × PAG of White classmates | .15 | -.09, .40 |  | -.04 | -.13, .04 |
| Peer victimization × PAG of Black classmates | .04 | -.14, .23 |  | .03 | -.02, .08 |
| Peer victimization × PSRC | -.24 | -.78, .29 |  | -.05 | -.21, .12 |
| PAG of White classmates × PSRC | -.48^†^ | -1.03, .07 |  | .20^*^ | .02, .37 |
| PAG of Black classmates × PSRC | .20 | -.16, .56 |  | -.07 | -.21, .07 |
| PAG of White classmates × Peer victimization × PSRC | .69 | -.18, 1.55 |  | -.42^***^ | -.66, -.19 |
| PAG of Black classmates × Peer victimization × PSRC | -.09 | -.74, .55 |  | -.03 | -.21, .16 |

*Note.* PAG = perceived aggression. PSRC = percentage same-race classmates.

^†^*p* < .10. ^*^ *p* < .05. ^**^ *p* < .01. ^***^ *p* < .001.

Table S21

*Parameter Estimates and 95% Confidence Intervals for Anxiety Latent Growth Curve Model – White Children*

|  | Anxiety intercept | |  | Anxiety slope | |
| --- | --- | --- | --- | --- | --- |
| Predictor | *b* | 95% CI |  | *b* | 95% CI |
| Peer victimization | .06 | -.04, .16 |  | .02 | -.03, .06 |
| PAG of White classmates | -.08 | -.18, .02 |  | .03 | -.01, .08 |
| PAG of Black classmates | .06 | -.01, .12 |  | .00 | -.04, .03 |
| PSRC | .11 | -.21, .43 |  | -.01 | -.12, .10 |
| Peer victimization × PAG of White classmates | .05 | -.14, .24 |  | -.14^**^ | -.23, -.05 |
| Peer victimization × PAG of Black classmates | .07 | -.09, .23 |  | .13^***^ | .07, .20 |
| Peer victimization × PSRC | -.37 | -.91, .17 |  | .11 | -.19, .40 |
| PAG of White classmates × PSRC | -.26 | -1.12, .60 |  | -.10 | -.34, .15 |
| PAG of Black classmates × PSRC | .22 | -.37, .81 |  | .07 | -.10, .25 |
| PAG of White classmates × Peer victimization × PSRC | .01 | -.97, 1.00 |  | -.51^**^ | -.82, -.20 |
| PAG of Black classmates × Peer victimization × PSRC | .27 | -.42, .95 |  | .08 | -.21, .37 |

*Note.* PAG = perceived aggression. PSRC = percentage same-race classmates.

^†^*p* < .10. ^*^ *p* < .05. ^**^ *p* < .01. ^***^ *p* < .001.

Table S22

*Parameter Estimates and 95% Confidence Intervals for Social Withdrawal Latent Growth Curve Model – White Children*

|  | Social withdrawal intercept | |  | Social withdrawal slope | |
| --- | --- | --- | --- | --- | --- |
| Predictor | *b* | 95% CI |  | *b* | 95% CI |
| Peer victimization | .18^***^ | .11, .26 |  | -.01 | -.05, .04 |
| PAG of White classmates | .01 | -.06, .08 |  | .00 | -.04, .04 |
| PAG of Black classmates | -.01 | -.06, .03 |  | .00 | -.02, .02 |
| PSRC | -.09 | -.37, .20 |  | -.01 | -.09, .08 |
| Peer victimization × PAG of White classmates | -.05 | -.29, .18 |  | .01 | -.08, .09 |
| Peer victimization × PAG of Black classmates | .17 | -.06, .39 |  | -.03 | -.09, .04 |
| Peer victimization × PSRC | -.22 | -.77, .33 |  | .01 | -.25, .27 |
| PAG of White classmates × PSRC | .21 | -.44, .86 |  | -.26^†^ | -.53, .001 |
| PAG of Black classmates × PSRC | -.17 | -.55, .21 |  | .25^**^ | .10, .39 |
| PAG of White classmates × Peer victimization × PSRC | .31 | -.49, 1.11 |  | .01 | -.23, .26 |
| PAG of Black classmates × Peer victimization × PSRC | -.31 | -1.01, .39 |  | .19 | -.09, .46 |

*Note.* PAG = perceived aggression. PSRC = percentage same-race classmates.

^†^*p* < .10. ^*^ *p* < .05. ^**^ *p* < .01. ^***^ *p* < .001.

Table S23

*Parameter Estimates and 95% Confidence Intervals for Aggressive Behavior Latent Growth Curve Model – White Children*

|  | Aggressive behavior intercept | |  | Aggressive behavior slope | |
| --- | --- | --- | --- | --- | --- |
| Predictor | *b* | 95% CI |  | *b* | 95% CI |
| Peer victimization | 1.01^***^ | -.93, 1.09 |  | -.06^*^ | -.11, -.01 |
| PAG of White classmates | .11^***^ | .05, .16 |  | -.02 | -.06, .01 |
| PAG of Black classmates | -.06^**^ | -.11, -.02 |  | .03^*^ | .004, .06 |
| PSRC | .10 | -.07, .26 |  | -.02 | -.13, .10 |
| Peer victimization × PAG of White classmates | .07 | -.11, .25 |  | -.05 | -.16, .07 |
| Peer victimization × PAG of Black classmates | -.22^**^ | -.39, -.06 |  | .02 | -.07, .12 |
| Peer victimization × PSRC | .30 | -.09, .68 |  | .11 | -.13, .36 |
| PAG of White classmates × PSRC | -.16 | -.60, .27 |  | -.15 | -.36, .06 |
| PAG of Black classmates × PSRC | .04 | -.29, .38 |  | .03 | -.16, .21 |
| PAG of White classmates × Peer victimization × PSRC | .75^**^ | .26, 1.24 |  | -.14 | -.49, .21 |
| PAG of Black classmates × Peer victimization × PSRC | -.93^***^ | -1.34, -.52 |  | .25^†^ | -.004, .50 |

*Note.* PAG = perceived aggression. PSRC = percentage same-race classmates.

^†^*p* < .10. ^*^ *p* < .05. ^**^ *p* < .01. ^***^ *p* < .001.

Table S24

*Parameter Estimates and 95% Confidence Intervals for Prosocial Behavior Latent Growth Curve Model – White Children*

|  | Prosocial behavior intercept | |  | Prosocial behavior slope | |
| --- | --- | --- | --- | --- | --- |
| Predictor | *b* | 95% CI |  | *b* | 95% CI |
| Peer victimization | -.88^***^ | -1.01, -.75 |  | -.04 | -.14, .07 |
| PAG of White classmates | .01 | -.10, .12 |  | -.05 | -.13, .03 |
| PAG of Black classmates | .03 | -.05, .10 |  | -.03 | -.07, .01 |
| PSRC | .14 | -.19, .46 |  | -.09 | -.33, .16 |
| Peer victimization × PAG of White classmates | -.16 | -.41, .08 |  | .13 | -.06, .31 |
| Peer victimization × PAG of Black classmates | .22^*^ | .03, .40 |  | -.04 | -.16, .08 |
| Peer victimization × PSRC | -.94^*^ | -1.67, -.22 |  | .33 | -.16, .82 |
| PAG of White classmates × PSRC | -.37 | -1.07, .34 |  | .08 | -.41, .57 |
| PAG of Black classmates × PSRC | -.01 | -.48, .47 |  | .20 | -.12, .52 |
| PAG of White classmates × Peer victimization × PSRC | .01 | -.85, .86 |  | -.28 | -.89, .32 |
| PAG of Black classmates × Peer victimization × PSRC | .67^*^ | .05, 1.29 |  | -.54^**^ | -.94, -.15 |

*Note.* PAG = perceived aggression. PSRC = percentage same-race classmates.

^†^*p* < .10. ^*^ *p* < .05. ^**^ *p* < .01. ^***^ *p* < .001.

**Peer-reported Prosocial Behavior - Black Children**

There was a negative perceived aggression of Black peers × percent same-race classmates interaction on the slope of peer-reported prosocial behavior (-.23, *p* = .04). Perceived aggression of black peers was negatively associated with the peer reported prosocial behavior slope when Black children were in a high percent same-race classroom (-.11, *p* = .02) but was not associated with the slope when in a low percent same-race classroom (.01, *p* = .83). Estimated trajectories are presented in Figure S1 below. As can be seen, at high percent same-race classmates, Black children evidenced a marginally significant increase in peer-reported prosocial behavior across the school year when Black children viewed their Black peers as low in aggression (.11, *p* = .09), and a nonsignificant decrease in the in peer-reported prosocial behavior when they viewed their Black peers as high in aggression (-.05, *p* = .37). There were no significant effects of perceiving Black classmates as aggressive at high or low levels of PSRC in the fall or spring.

*Figure S1*

*p* = .51

*p* = .08

*p* = .21

*p* = .25

Slope = .11, *p* = .09

**Depression - White Children**

There was a significant positive peer victimization × perceived aggression of White classmates × PSRC interaction on the slope. Peer victimization was marginally negatively associated with changes in depression when White children had a high PSRC who they viewed as high in aggression (-.06, *p* = .07), but was not associated with changes in depression when they had a low PSRC who they viewed as low (-.01, *p* = .87) or high (.01, *p* = .57) in aggression or when they had a high PSRC who they viewed as low in aggression (.04, *p* = .34). Estimated trajectories are presented in Figure S2 in the supplement. A significant increase in depression (.04, *p* = .007) was found when White children experienced low levels of peer victimization and had a high PSRC who they viewed as high in aggression. This increase did not lead to meaningful effects in the spring beyond the positive main effect of peer victimization.

*Figure S2*

a

a

*p* < .001

*p* = .06

*p* = .28

Slope = .04, *p* = .007

*p* < .001

*p* < .001

*p* < .001

(a) Low Percentage Same-Race Classmates (b) High Percentage Same-Race Classmates

^a^ There was a main effect of peer victimization on the intercept in the fall and spring. There were no significant interactions. Simple slopes at high and low levels of perceived white aggression are presented for descriptive purposes.

*Figure S3*

*Estimated Trajectories of Anxiety at High and Low Levels of Peer Victimization and Perceived White Classmates’ Aggression at a high and low PSRC – White Children*

1. Low Percentage Same-Race Classmates

*^p^* ^=.23^

*^p^* ^= .20^

1. High Percentage Same-Race Classmates

*Note.* PSRC = percentage same-race classmates. Agg = behavior.

**Social Withdrawal - White Children**

Perceived aggression of Black peers was positively associated with the slope of withdrawal when White children had a high percentage of same-race classmates (.04, *p* = .01) but was negatively associated with the slope of withdrawal when White children had a low percentage of same-race classmates (-.04, *p* = .02). Estimated trajectories are presented in Figure S4. Although there were differences in slopes as a function of perception of Black peer’s aggression and percent same-race classmates, none of these slopes were significantly different from zero.

*Figure S4*

*^p^* ^= .53^

*^p^* ^= .50^

*Note.* PSRC = percentage same-race classmates. Agg = behavior. All slopes significant at *p* = .08.

*Figure S5*

*Estimated Trajectories of Aggression at High and Low Levels of Peer Victimization and Perceived White Classmates’ Aggression at a High and Low PSRC – White Children*

*^p^* ^< .001^

*^p^* ^< .001^

1. Low PSRC

*^p^* ^< .001^

*^p^* ^< .001^

^Slope = .05,^ *^p^* ^= .04^

^Slope = .06,^ *^p^* ^= .03^

1. Low PSRC

*Note.* PSRC = percentage same-race classmates. Agg = behavior.

*Figure S6*

*Estimated Trajectories of Aggression at High and Low Levels of Peer Victimization and Perceived Black Classmates’ Aggression at a High and Low PSRC – White Children*

^Slope = .08^ *^p^* ^< .001^

*^p^* ^< .001^

*^p^* ^< .001^

1. Low PSRC

^Slope = .06,^ *^p^* ^= .02^

^Slope = .05^ *^p^* ^= .04^

*^p^* ^< .001^

*^p^* ^< .001^

*^p^* ^< .001^

1. High PSRC

*Note.* PSRC = percentage same-race classmates. Agg = behavior.

*Figure S7*

*Estimated Trajectories of Aggression at High and Low Levels of Peer Victimization and Perceived Black Classmates’ Prosocial Behavior at a High and Low PSRC – White Children*

^Slope = -.08,^ *^p^* ^= .01^

^a^

*^p^* ^< .001^

*^p^* ^< .001^

*^p^* ^< .001^

1. Low PSRC

*^p^* ^< .001^

*^p^* ^< .001^

*^p^* ^< .001^

^Slope = -.08,^ *^p^* ^= .04^

1. High PSRC

*Note.* PSRC = percentage same-race classmates. Agg = behavior.

^a^ At low PSRC, by the spring, the peer victimization × perceived Black classmates’ aggression was significant (.20, *p* = .02).
